# The influence of the forest corridors to the north of the Andes on the diversification of the bright‐rumped Attila, *Attila spadiceus* (Passeriformes, Tyrannidae), during the climatic oscillations of the middle Pleistocene

**DOI:** 10.1002/ece3.70331

**Published:** 2025-01-21

**Authors:** Patrícia Mendonça, Lincoln Silva Carneiro, Victor Leandro‐Silva, Alexandre Aleixo, Juliana Araripe, Péricles S. Rêgo

**Affiliations:** ^1^ Laboratory of Genetics and Conservation, Institute of Coastal Studies Universidade Federal do Pará Bragança Pará Brazil; ^2^ Laboratory of Ornithology and Molecular Biology Museu Paraense Emílio Goeldi/Universidade Federal do Pará Belém Brazil; ^3^ Laboratory of Bird Ecology and Evolution Universidade Federal de Pernambuco Recife Brazil; ^4^ Instituto Tecnológico Vale Belém Pará Brazil

**Keywords:** Amazonia, Atlantic forest, birds, multilocus analysis, Neotropical region Phylogeography

## Abstract

This study aims to enhance our understanding of the temporal and spatial processes scales governing the evolutionary diversification of Neotropical birds with Trans‐ and Cis‐Andean populations of the species *Attila spadiceus* from South and Central America. Through a multilocus analysis of the mitochondrial (CytB and ND2) and nuclear genes (I7BF, I5BF, and G3PDH) of 41 samples representing six subspecies, we describe the existing molecular lineages of *A. spadiceus*, and estimate their demographic dynamics. We used Ecological Niche Modeling (ENM) with six different algorithms to predict the potential distribution of *A. spadiceus* in both present‐day and past scenarios, examining the overlap climatic niche between Cis‐ and Trans‐Andean lineages. The analysis confirms a relatively recent divergence of the Trans‐ and Cis‐Andean lineages, at approximately 0.25 million years ago (Ma). The niche modeling supports the existence of a dynamic scenario of the expansion and retraction of forest corridors in northwestern South America during the last glaciation. This suggests that the earlier orogenesis of the Andes was not a primary determinant of this dichotomy. Additionally, the analysis of population dynamics indicated a trend of increasing population size starting at 0.05 Ma for both lineages. Our findings highlight the significance of Pleistocene Forest corridors north of the Andes as the key factor maintaining communication before the separation of the lineages, likely associated with the retraction of this forest. We highlight the absence of any significant differentiation between the disjunct Amazonian and Atlantic Forest populations, at both part of the Cis‐Andean lineage. The phylogeographic profile of *A. spadiceus* diverges from the patterns observed in other Neotropical birds, which emphasizes the need for further research on the role of the forest corridors of the northern Andes as drivers of diversification, to provide comprehensive insights into the processes that led to the formation of the region's present‐day avian diversity.

## Graphical abstract

Our findings highlight the significance of Pleistocene Forest corridors north of the Andes as the key factor maintaining communication before the separation of the lineages of *A. spadiceus*. We highlight the absence of any significant differentiation between the disjunct Amazonian and Atlantic Forest populations, both part of the Cis‐Andean lineage. The phylogeographic profile of *A. spadiceus* diverges from the patterns observed in other Neotropical birds.

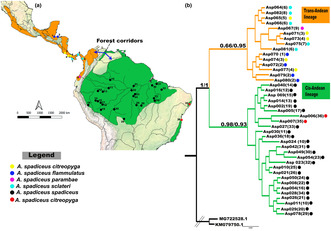

## 1 INTRODUCTION

The present‐day configuration of the biodiversity of the Neotropical region has been influenced fundamentally by vicariant events, such as the formation of the isthmuses of Panama (Coates & Stallard, 2013) and Tehuantepec (IT) (Barrier et al., 1998), the Andes (Gregory‐Wodzicki, 2000), and the drainage of the Amazon River (Hoorn et al., 2010; Ribas et al., 2011). The dispersal, retraction, and differentiation of the populations of this biota have been amply documented in studies that have revealed significant shifts in the occurrence and geographic distribution of these populations over time (Castillo‐Chora et al., 2021; D'Horta et al., 2013; Fernandes et al., 2014; Mendonça et al., 2022; Weir & Price, 2011). Species that have disjunct Trans‐ and Cis‐Andean populations (to the west and east of the Andes, respectively) are one example of the results of the historical processes that have molded the region's biogeography. These species provide excellent models for the understanding of phylogeographic processes and, in particular, the adaptation of a species to distinct environments, its evolutionary history, and population dynamics. Thus, the historical processes that shaped the biogeography of the region include geographical separation caused by tectonic (Lavinia et al., 2015) and climatic (Mendonça et al., 2022; Weir & Price, 2011) events.

For many widely distributed bird species, the orogenesis of the Andes resulted in the disjunct distribution of populations to the east and west of this mountain range. A few studies have focused on the role of the Andes as a primary physical barrier, which reduces gene flow and causes divergence through vicariance (D'Horta et al., 2013; Fernandes et al., 2014; Lavinia et al., 2015; Reis et al., 2019), as well as its secondary or even contingent role, in the maintenance of this divergence (Reis et al., 2019; Ribas et al., 2007). In the latter case, in addition to the indirect influence of the Andes, the varying estimates of the divergence times of avian lineages during the Pleistocene indicate that much of the region's biota was also impacted by climatic oscillations. These oscillations alternatively favored the formation of forest corridors across a porous region to the north of the Andes. This may have contributed to the connectivity of populations in Central and South America (Alfaro et al., 2015; Haffer, 1967; Mendonça et al., 2022; Weir & Price, 2011).

**FIGURE 1:**
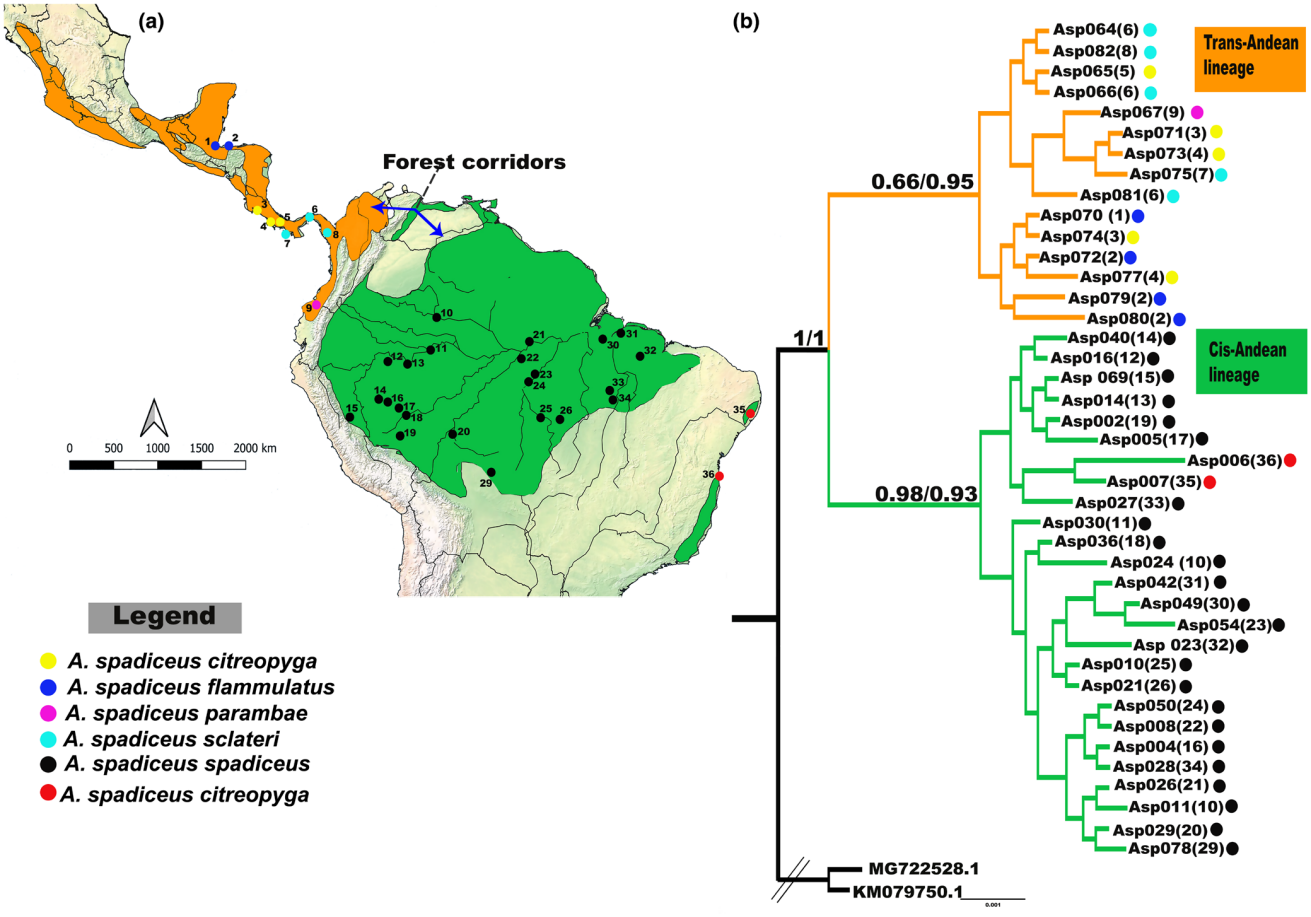
(a) Distribution of the *A. spadiceus* sample localities within the ranges of the two lineages recovered here by Bayesian Inference (BI). The two lineages are color coded (orange = Trans‐Andean, green = Cis‐Andean), as are the localities of the six subspecies analyzed here (see legend). (b) The BI topology recovered for the multilocus (support values on the left of the bar) and mitochondrial (support values on the right of the bar) datasets. The number of each sampling locality is given within parentheses after the sample code. Highlighted supports were those with greater relevance in demonstrating the separation of the two lineages. Probabilities ≥ 0.95 are highly supported. The branch of the external group has been truncated (//) to facilitate the presentation of the results.

The Bright‐rumped Attila, *Attila spadiceus* (Passeriformes, Tyrannidae) (Gmelin, 1789), is amply distributed in the Neotropical region, where it occupies a number of different forest environments (Walther et al., 2020). Comprising a total of 12 subspecies (Figure 1a), *A. spadiceus* is an interesting group for the evaluation of these biogeographic hypotheses. The principal phylogeographic analyses of this species were published by Smith et al. (2014) and Mendonça et al. (2022), both of which were based exclusively on mitochondrial markers, and identified two clades, one Trans‐Andean and the other, Cis‐Andean. Given these findings, we sought to refine our understanding of the mechanisms that drove Neotropical avian diversity during the Pleistocene, at both temporal and spatial scales, and in particular, identify the elements of the evolutionary history of *A. spadiceus* that underpinned its current profile of intraspecific diversification and distribution in different environments. For this, we developed a multilocus molecular approach to analyze the two lineages, and verify the role of the Andean Forest corridors in the formation of the two groups recovered in these previous matrilineal studies, as well as defining their demographic history.

## 2 MATERIALS AND METHODS

### 2.1 Sampling

The present study was based on the analysis of 41 samples of muscle tissue, obtained from *A. spadiceus* specimens collected from both Trans‐ and Cis‐Andean populations (Figure 1a and Appendix S1, Supporting Information, Table S1). The Ornithology Sector of the Museu Paraense Emílio Goeldi (MPEG) provided 24 specimens, representing two of the recognized subspecies, one from the Amazon biome (*A. s. spadiceus*, *n =* 22) and the other from the Atlantic Forest (*A. s. uropygiatus*, *n =* 2). We obtained an additional 17 samples from the Louisiana State University Museum of Natural Science (LSUMZ), which represented a further five subspecies: *A. s. citreopyga* (*n* = 4), *A. s. flammulatus* (*n* = 5), *A. s. parambae* (*n* = 1), *A. s. sclateri* (*n* = 5), and *A. s. spadiceus* (*n* = 2).

### 2.2 Laboratory procedures and edition of the sequences

We extracted the DNA from the samples using the Wizard® Genomic Purification kit (Promega). We evaluated the extracted DNA by electrophoresis in 1% agarose gel, followed by visualization under an ultraviolet transilluminator.

We amplified five genetic markers by Polymerase Chain Reaction (PCR), including two mitochondrial genes – NADH deshydrogenase subunit 2 (ND2) and Cytochrome *b* (CytB) – and three nuclear regions: introns 5 (I5BF) and 7 (I7BF) of the ß‐fibrinogen gene, and Glyceraldehyde 3‐phosphate dehydrogenase (G3PDH). The primers used to extract these five sequences are summarized in (Appendix S2, Supporting Information, Table S2). The amplification conditions for each gene region can be found in the respective references (Appendix S2, Supporting Information, Table S2). We then sequenced the samples using Sanger et al.'s (1977) dideoxy thermal method. We obtained parts of the fragment of the CytB gene from GenBank, for which the accession numbers are given in (Appendix S1, Supporting Information, Table S1). The newly generated sequences will be made available in GenBank along with their corresponding accession numbers. We determined the nucleotide sequences of the other fragments obtained from the sequencing reaction in an ABI 3500 automatic sequencer (Applied Biosystems).

We visualized and corrected the nucleotide sequences obtained here in BioEdit 7.2 (Hall, 1999), and aligned the multiple sequences automatically in CLUSTAL‐W (Thompson et al., 1997), which was also run in BioEdit. We confirmed that the double peaks observed in the nuclear markers were heterozygous sites, and codified them according to their IUPAC nucleotide codes. We estimated the gametic phases of the nuclear markers using the Bayesian approach, in the PHASE algorithm (Stephens & Donnelly, 2003) which was implemented in DNAsp (Librado & Rozas, 2009). We considered the gametic phases with a probability of at least 0.6 to be resolved (Harrigan et al., 2008) and excluded those with smaller values from the analysis.

### 2.3 Phylogenetic analyses

To construct the phylogenetic arrangements derived from Bayesian Inference (BI), we concatenated the genes into two datasets in Sequence Matrix 1.7.8 (Vaidya et al., 2001). Dataset 1 encompassed all the concatenated sequences, that is, G3PDH, I7BF, I5BF, ND2, and CytB, while dataset 2 included only the mitochondrial markers (ND2 and CytB). We identified the most adequate nucleotide substitution model and the optimal partitioning scheme (gene or codon) for the data in PartitionFinder 2.1 (Lanfear et al., 2016).

We generated two Bayesian Inference topologies in MrBayes 3.2.7 (Ronquist et al., 2012), one with the complete multilocus database, in which two partitions were indicated (see Appendix S3, Supporting Information, Table S3), and rooted by *Attila cinnamomeus*. The other topology was recovered from the mitochondrial data (ND2 and CytB), with three partitions (see Appendix S3, Supporting Information, Table S3), rooted by *Attila torridus*, based on the sequences available from GenBank. We ran these analyses on the CIPRES platform (Miller et al., 2010), based on two runs of the Markov Chain Monte Carlo (MCMC). We generated the multilocus topology through 30 million generations, to provide 10,000 trees, of which, (10%) were discarded as burn‐in. We generated the mitochondrial topology through 5 million generations, which provided 10 ka trees, with (10%) being discarded as burn‐in.

### 2.4 Estimates of divergence time

We used the five sequences obtained in the present study to produce a species tree, based on the Bayesian coalescence approach, which we ran in BEAST 1.8 (Drummond et al., 2012). We partitioned the data per gene in PartitionFinder 2.1 (Lanfear et al., 2016). We generated an input file (.xml) in BEAUTi, which we submitted to a control run, and then selected the relaxed clock and uncorrelated lognormal distribution for all the genes. We then evaluated the ucld.stdev values, which were close to zero for the ND2, CytB, G3PDH, and I7BF markers, which indicates that the hypothesis of a uniform substitution rate could not be rejected. Given this, we applied the strict clock for the subsequent runs, and then implemented two independent runs of 130 ka generations, with a burn‐in of 10%. For these analyses, we used a mutation rate of 2.1% per million years for the CytB marker (Weir & Schluter, 2008) and 2.5% per million years for the ND2 (Smith & Klicka, 2010), and estimated the other markers in BEAST 1.8. We verified the convergence of the chains in TRACER 1.7.1 (Rambaut et al., 2018), assuming ESS values of over 200.

### 2.5 Population structure and demographic history

We determined the haplotypes through their phased sequences, in DNAsp 5.10.1 (Librado & Rozas, 2009), and compiled the haplotype networks in Haploviewer (Salzburger et al., 2011), based on the maximum likelihood probabilities, which were calculated in Phylip 3.6 (Felsenstein, 2004). We ran neutrality tests to evaluate the historical changes in the groups of specimens defined in the analyses, and to verify whether the populations had undergone demographic expansion, equilibrium or decline, based on Fu's *Fs* (Fu, 1997) and Tajima's *D* (Tajima, 1989), run in Dnasp 5.10 (Librado & Rozas, 2009). We calculated the genetic distance for each marker within and between the lineages (uncorrected *p* distances) in MEGAX (Kumar et al., 2018), using the lineages recovered by the Bayesian Inference as the groups.

We estimated the changes in effective population size (*N*_*e*_) over time to determine the level of demographic variation in each *A. spadiceus* lineage. For this, we generated separate nucleotide substitution models for the different lineages of the multilocus database (see Appendix S3, Supporting Information, Table S3) in PartitionFinder 2.1 (Lanfear et al., 2016) and obtained Extended Bayesian Skyline Plots (EBSPs) for the lineages (Heled & Drummond, 2008) in BEAST 1.8 (Drummond et al., 2012).

### 2.6 Niche modeling and overlap

We used Ecological Niche Modeling (ENM) to predict the potential distribution of *A. spadiceus* in both the present day and past scenarios. This niche modeling approach verifies the association between environmental features and the known points of occurrence of the species, with the aim of determining the variables that mold the ecology of the species (Peterson et al., 2011). We used seven bioclimatic variables from PaleoClim (Brown et al., 2018) to characterize the environment, both present and past. These seven bioclimatic variables present low multicollinearity and were selected by Spearman correlation (*r* < 0.7) (See Table S4 for select variables). PaleoClim provides 19 bioclimatic variables for the present day and 10 different moments in the past, although we only used two of these past scenarios here: Last Glacial Maximum (LGM: ca. 21 ka) and the Last Interglacial (ca. 130 ka). The climate predictors have a resolution of 2.5 arc minutes (~4.5 × 4.5 km at the equator). We obtained a total of 4507 *A. spadiceus* occurrence records from field collections (Table S1), database (GBIF, 2024), and published papers. We then applied a spatial correlation in Sdmtoolbox (Brown, 2014), which left 2213 points for analysis. This correlation is applied until there is only one data point per cell, by removing repeated cells from the grid, together with those that contain the same environmental information. We mapped the occurrence records onto a cell grid.

To predict the current and past potential distribution of *A. spadiceus*, we used ecological niche modeling techniques (ENMs). Niche modeling estimates associations between environmental aspects (most often climate) and known species occurrences to characterize the range of conditions under which the species' populations survive, called environmental (or climatic) suitability (Franklin, 2009; Peterson et al., 2011). As the predictions will vary by the algorithms used to generate the models (Barry & Elith, 2006; Diniz‐Filho et al., 2009), we used six different algorithms for modeling the potential distribution of *A. spadiceus*. These algorithms were: (1) Bioclim (Nix, 1986), (2) GLM (Guisan et al., 2002), (3) Domain (Gower distance: Carpenter et al., 1993), (4) RandomForest (Liaw & Wiener, 2002), (5) Maximum Entropy – Maxent (Philips & Dudik, 2008), and (6) Support Vector Machines, SVM (Tax & Duin, 2004). All these algorithms were run in the “dismo” package (Hijmans et al., 2022) using the specific functions that correspond to each algorithm, except for SVM, which was run using the ksvm function of the “kernlab” package (Karatzoglou et al., 2004), and RandoForester, run using the specific function of the “randomForest” package (Liaw & Wiener, 2002). To calibrate the models based on presence‐background observations (i.e., SVM and Maxent), we selected the background points randomly across a grid of cells in South America (except for cells with the presence of the species) as the input for the evaluation of the models, as recommended in some previous studies (e.g., Sobral‐Souza et al., 2015; Thuiller et al., 2004).

Following an ensemble algorithmic approach (Araújo & New, 2007), we concatenated the binary maps produced by each replicate of each algorithm to calculate the frequency of predicted presences for each grid cell, producing a single consensus map per climate scenario. We used the maximum sensitivity and specificity limit recommended by Liu et al. (2016), that is, the lowest climate suitability value from an occurrence record (<0.41), as a cutoff limit for calculating predicted binary maps (a similar approach to Anunciação et al., 2023; Jiménez‐Valverde et al., 2011; Luna et al., 2022; Peres et al., 2015; Sobral‐Souza et al., 2015).

We generated a total of 20 replicates for each of the models, which we adjusted by a double‐partition criterion (75% and 25%) for the general modeling of the species through training models and the evaluation of their performance. We selected the 75% and 25% points randomly for each of the 20 replicates in each algorithm. These repetitions are carried out to reduce the spatial structure between the training and test data sets, which provides less biased evaluations. We used the True Skill Statistic (TSS) – which ranges from −1 to 1 – to evaluate the models. In this approach, negative values or values close to zero indicate that the predictions of the model are no different from a configuration generated randomly, whereas models with values close to 1 are considered to be very good. In general, models with TSS values of over 0.5 are considered to be acceptable (Allouche et al., 2006).

We checked niche overlap between Trans and Cis‐Andean lineages using the EcoPast package (Broennimann et al., 2023). We used PCA‐env, Schoener's *D* (Schoener, 1968), and Warren's *I* (Warren et al., 2008) to quantify niche overlap between populations, based on niche equivalence and similarity. Schoener's *D* is based on direct measurements of modeled occurrence densities in environmental space, while Warren's *I* is based on the modified Hellinger distance used to compare two probability distributions. These two indices range from 0 (no similarity) to 1 (high similarity). We controlled for the effect of climate equivalence through a similarity test (Warren et al., 2008), which examines whether the niches of two populations with partial or non‐overlapping distributions are more different than randomly expected (Warren et al., 2008).

## 3 RESULTS

We obtained Bayesian Inference topologies based on each of the two datasets, that is, the complete multilocus sequence of 3401 base pairs (bp) and the concatenated mitochondrial sequences (CytB and ND2), with 1767 bp (Figure 1b). Both topologies revealed two principal clades, which were divided and maintained by the physical barrier formed by the Andes Cordillera. The first clade, denominated here as the Trans‐Andean lineage, which is located to the west of the Andes, is formed by four subspecies *A. s. citreopyga*, *A. s. flammulatus*, *A. s. parambae*, and *A. s. sclateri*. The second clade, the Cis‐Andean lineage, is located to the east of the Andes, and is composed of two subspecies, *A. s. spadiceus* and *A. s. uropygiatus* (Figure 1a,b).

**FIGURE 2:**
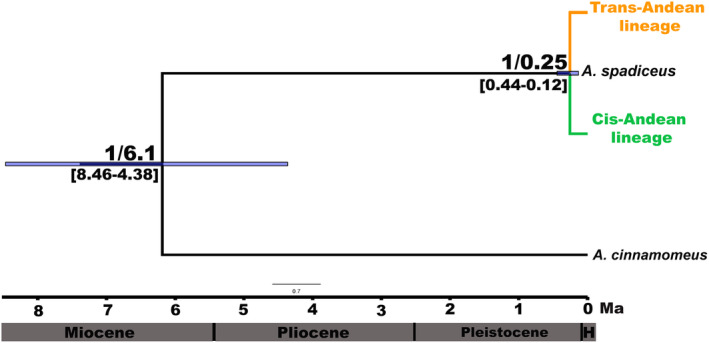
Species tree showing the coalescence time of the Trans‐Andean (east of the Andes) and Cis‐Andean (west of the Andes) lineages of *A. spadiceus*, which diversified at approximately 0.25 Ma. The values above the branches are the posterior probability/mean coalescence time (age of the node), while those below the branches are the confidence interval (95% Highest Posterior Density, or HPD) of the divergence time. The timeline of the geological epochs is shown at the bottom of the plot for reference (H = Holocene).

**FIGURE 3:**
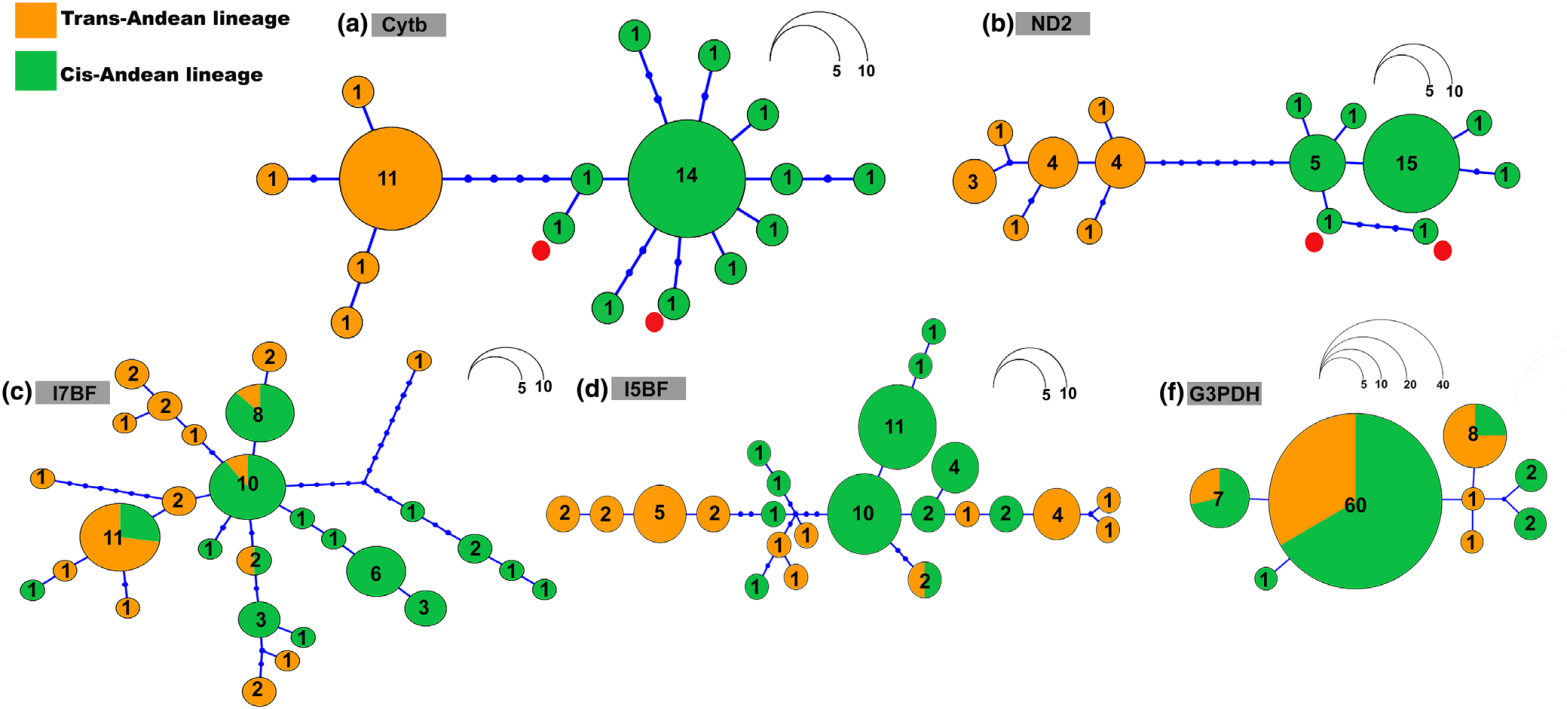
Haplotype networks of the five markers sequenced in the present study. The two lineages are color‐coded as in Figure 1, and the two Atlantic Forest samples are indicated by the red dots in the mitochondrial networks.

The species tree further reinforces this division into two principal lineages, with absolute statistical support (pp = 1), which coincides with the BI topology (Figure 2). The two lineages diverged at some time within the estimated Confidence Interval, that is, between 0.12 and 0.44 Ma, with a mean estimate of 0.25 Ma. We also recovered two haplogroups in the haplotype networks of each of the mitochondrial markers (Figure 3a,b), which is, once again, consistent with the BI topology. None of the haplotypes were shared between samples from the two lineages in either of these networks. By contrast, the nuclear markers did not generate well‐defined haplogroups, given that haplotypes were shared between individuals from the two different lineages defined by the BI topology.

As the existence of the two clades was well established in these analyses, we calculated the divergence rates between and within these two groups. Divergence (uncorrected *p* distances) within Trans‐Andean lineage ranged from 0.08% (recovered from the CytB marker) to 0.036% (I5BF). Within the Cis‐Andean lineage, divergence varied from 0.04% (for G3PDH) to 0.34% (I7BF). The divergence between the two lineages was greater in the mitochondrial markers, peaking at 1.09% in the case of ND2, although much lower values were recorded for the nuclear markers, reaching 0.05% in G3PDH (Table 1).

**TABLE 1:** Uncorrected genetic divergence (*p* distance) of each gene sequenced in the present study, within and between the Trans‐Andean and Cis‐Andean lineages.

Lineage	Uncorrected *p* distance recorded for the marker				
	CytB	ND2	I5BF	I7BF	G3PDH
Trans‐Andean	0.08%	0.21%	0.36%	0.18%	0.11%
Cis‐Andean	0.16%	0.13%	0.12%	0.34%	0.04%
Trans‐Andean vs. Cis‐Andean	0.84%	1.09%	0.22%	0.28%	0.05%

**FIGURE 4:**
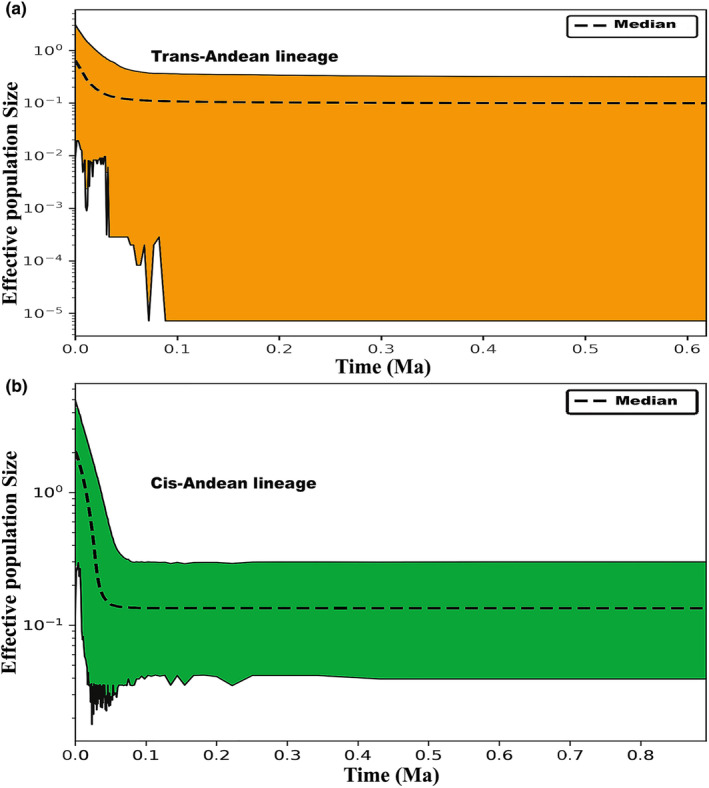
Extended Bayesian Skyline Plots (EBSP) based on the five molecular markers sequenced in the present study (see specific genes listed in APPENDIX 2, Table S2), showing the variation in the estimates of effective population size of the two *A. spadiceus* lineages, (a) trans‐Andean lineage and (b) cis‐Andean lineage (color‐coded as in the previous figures) over time. The dashed line shows the median estimate, while the solid lines correspond to the 95% Confidence Interval. The x‐axis shows the time in millions of years before the present day, which is at zero (0).

The demographic history of the lineages is represented by the Extended Bayesian Skyline Plots (Figure 4a,b), which indicate a clear pattern of growth in the effective size of the populations of both lineages from approximately 0.05 Ma to the present day. In the neutrality tests (Table 2), negative and significant results were found for mitochondrial markers only in the Cis‐Andean lineage, which are consistent with a recent tendency for population expansion.

**TABLE 2:** The values of Fu's *Fs* and Tajima's *D* recorded for the five markers sequenced in the Trans‐Andean and Cis‐Andean *A. spadiceus* lineages.

	Value recorded for the marker				
	ND2	CytB	I7BF	I5BF	G3PDH
Trans‐Andean lineage					
Fu's *F*s	−1.853	−1.287	−5.241	−3.264	−1.093
Tajima's *D*	−0.66053	−1.51811	−0.14666	1.03726	−0.71732
Cis‐Andean lineage					
Fu's *Fs*	−3.151	−7.449	−2.738	−4.765	−4.765
Tajima's *D*	−**1.81219**	**−2.21646**	0.13613	−1.15078	−1.31816

*Note*: The significant (*p* < .005) values are shown in bold type.

**FIGURE 5:**
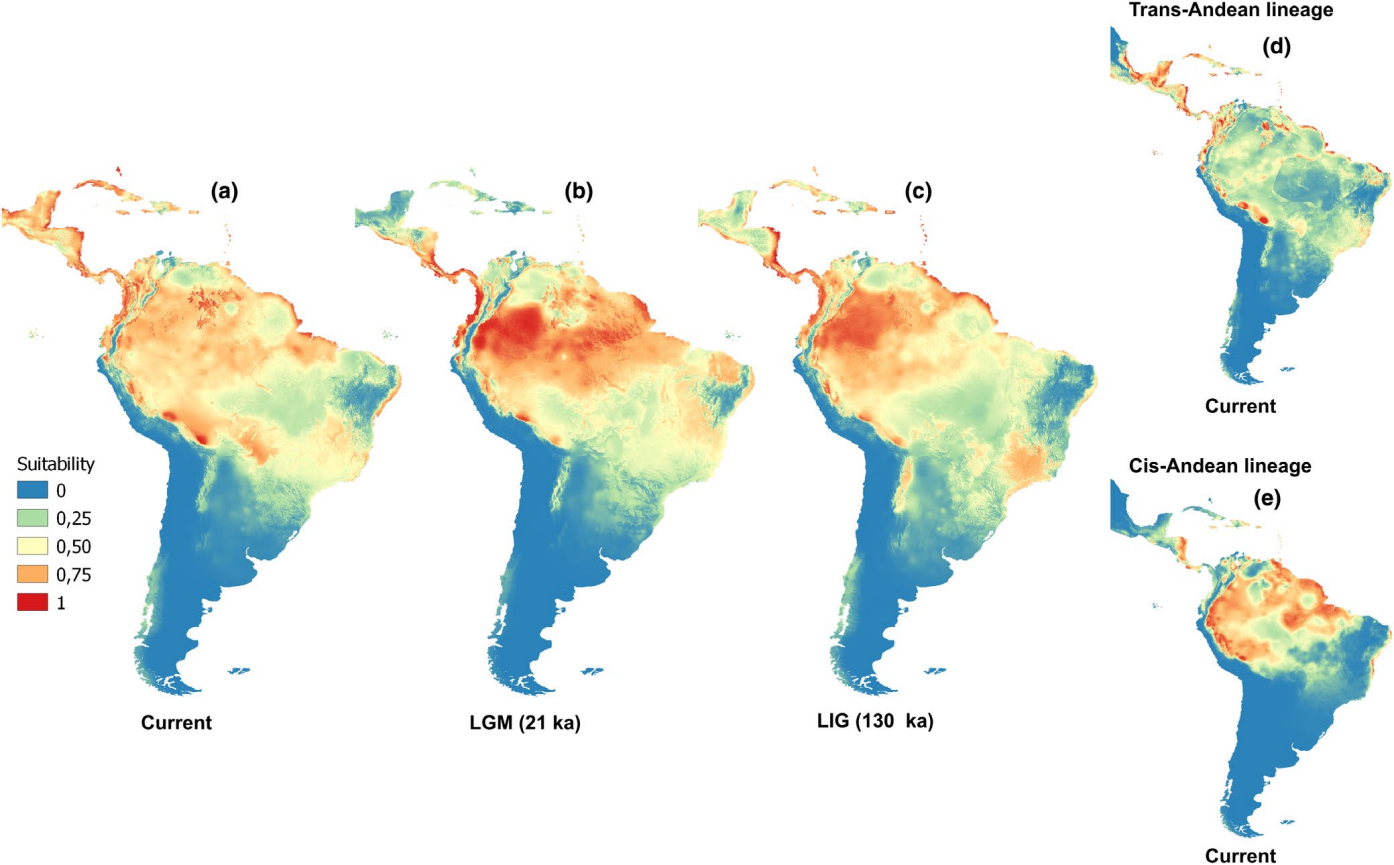
Niche suitability model of *Attila spadiceus* under three different temporal climatic scenarios (a–c) and current scenarios of the Cis‐ and Trans‐Andean lineages (d, e, see legend). The areas in red have a highly suitable climate, while the climate of the areas along the blue gradient are increasingly unsuitable.

The evaluation of the six niche algorithms presented AUC values of less than 0.9 and TSS of 0.75, indicating an excellent model performance. The predictions of the temporal climate niche model indicate small contractions and expansions of the areas suitable for the species. Areas of stable climate in the neighboring contact zones between the two lineages can be found in distinct temporal scenarios, which indicates that, during the LGM, these two lineages would both have had access to climatic corridors (Figure 5a–e). The niche models generated for the two lineages indicated differentiation due to a lack of niche sharing (see Appendix S5 in Supporting Information, Figure S1). The results of the niche overlap analysis revealed high values (Schoener's *D* = 0.46 and Warren's *I* = 0.67), reinforcing the existence of marginally significant differences between the niches of the two lineages (see Appendix S6 in Supporting Information, Figure S3a,b and Appendix S4, Table S4). The PCA‐env also indicates a high degree of overlap in environmental space between the two populations (see Appendix S6 in Supporting Information, Figure S2b). The similarity and equivalence tests also showed high *D* values when comparing the two lineages.

## 4 DISCUSSION

### 4.1 Influence of the forest corridors to the north of the Andes on the diversification of *Attila spadiceus*

The multilocus analysis presented here confirmed the pattern of two *A. spadiceus* lineages distributed on opposite sides of the Andes, confirming the mitochondrial proposals of Smith et al. (2014) and Mendonça et al. (2022). Our results support a scenario in which the remnants of the Trans‐Andean *A. spadiceus* population to the east of the IT (Figure 1a), maintained contact with individuals from the Cis‐Andean lineage up until the middle Pleistocene. That is, the Chibanian stage (ICS, 2023), which is much later than the completion of the orogenesis of the Andes, around 2–5 million years ago through the north of Colombia (Gregory‐Wodzicki, 2000).

These populations were probably connected by forest corridors, which formed in the vicinity of the northern Andes, during the dynamic climatic scenario of the Pleistocene (Haffer, 1967; Weir & Price, 2011). These forested areas probably expanded and retracted successively over the course of the Pleistocene, resulting in a dynamic pattern of connection between the Trans‐ and Cis‐Andean populations. Haffer (1967) suggested that the humid vegetation forming these corridors retracted southward, interrupting their connectivity formations. This left evidence of ruptures at distinct moments in the evolutionary history of a number of different groups of animals (Alfaro et al., 2015; Weir & Price, 2011), which only diverged after the complete formation of the Andes (Gregory‐Wodzicki, 2000).

The dynamic configuration of these forest corridors is supported by our predicted climate scenarios for *A. spadiceus*, which indicate that, during the LGM, climatically adequate areas in northwestern South America would have been connected, albeit with subsequent retractions and ruptures in the corridors. One other piece of evidence that supports the differentiation of the two lineages is the oscillations in the climate scenarios that were observed when the lineages were evaluated separately (see Appendix S5 in Supporting Information, Figure S1), despite the reduced significance of the differences between their niches (see Appendix S6 in Supporting Information, Figure S2a,b).

The divergence times of the lineages recovered in the present study reflect the importance of these forest corridors. They were crucial for the maintenance of exchanges between the lineages right up until their separation, which may have occurred due to the retraction of these corridors. However, there is a possibility of post‐divergence gene flow between lineages, supported by the nuclear markers, which deserves further evaluation, given that the incomplete separation of lineages cannot be ruled out for this group. In other groups, such as the woodcreepers of the genus *Dendrocincla*, the retraction and expansion of the forest corridors mediated successive divergence events, the first of which occurred around 2.1 Ma, originating the Trans‐Andean species *Dendrocincla anabatina*, while the second was at approximately 0.9 Ma, when the Trans‐ and Cis‐Andean populations of *Dendocincla fuliginosa* were formed (Weir & Price, 2011). A late pattern of dispersal following the complete formation of the Andes has also been proposed for the squirrel monkey, *Saimiri oerstedii*, in Central America. This species was isolated from the Amazonian members of the genus at around 1–0.8 Ma, due to the retraction of the forest and the increase in arid environments with dunes and savannas in the vicinity of the northern Andes, especially in the region of the Venezuelan Llanos (Alfaro et al., 2015).

The success of the processes of expansion or dispersal of *A. spadiceus* may also be related to the ability of this bird to inhabit a diversity of humid forest environments (Walter et al., 2020), which is favored by the conservative nature of its niche as shown by the results of the present study (see Appendix S6 in Supporting Information, Figure S2a,b). The fact that this species prefers the forest canopy likely also favored these processes, as has been observed in other groups of birds (Burney & Brumfield, 2009). Future studies that seek to understand and evaluate in more detail the species' ability to maintain itself in various humid forest environments could deepen the interpretations presented here.

### 4.2 Trans‐ and cis‐Andean lineages

Central America has a diverse landscape, including the Guatemala Highlands, the Talamanca Cordillera (Marshall & Liebherr, 2000), the Nicaragua Depression (D'Horta et al., 2013), and the basin of the Panama Canal (Castillo‐Chora et al., 2021), all of which contribute to the high levels of biodiversity of the Neotropical region. Our findings indicate that there are no well‐supported independent groups in the Trans‐Andean lineage, which is inconsistent with the subdivisions proposed for the region, based on the influence of physical barriers.

The lack of genetic groups in this clade contradicts the expected differentiation of the species across Central America, given the environmental pressures imposed by the region's distinct landscapes (Castillo‐Chora et al., 2021; Marshall & Liebherr, 2000; Rodríguez‐Gómez & Ornelas, 2018). Although these pressures may have contributed to the development of the phenotypic variation observed in the lineage, we did not observe any systematic correlation of this variation in the portions of the genome analyzed here (Figure 1b). The phylogeographic structure recovered here points to a pattern of diversification that contradicts the region's principal evolutionary events, although a similar pattern has been recorded in some previous studies (Bagley & Johnson, 2014; Lavinia et al., 2015; Rocha‐Méndez et al., 2018).

One other prominent feature of the findings of the present study is the apparent lack of influence of the principal drivers of diversification that have been described for Amazonian birds, such as the configuration of the hydrographic system, Pleistocene refugia, and environmental heterogeneity (Berv et al., 2021; Fernandes et al., 2014; Haffer, 1967; Hoorn et al., 2010; Ribas et al., 2011; Wallace, 1852). None of these factors appear to have determined any noticeable level of differentiation within the biome. A number of alternative hypotheses have attempted to account for the biogeographic complexity of Amazonia and the specific responses of some species to the drivers of diversification in this region (Johnson et al., 2023; Silva et al., 2019; Smith et al., 2014). We suggest that the distinct profile observed in the Cis‐Andean lineage may reflect certain idiosyncratic characteristics, as observed in some other birds (Johnson et al., 2023). Yamaguchi (2022) postulated that the populations of some species ecologically adapted for dispersal may not be structured by geographic barriers. In this case, our evidence of a single genetic group may be linked to the ecological flexibility of the taxon in occupying humid forests, which may allow it to overcome physical barriers and environmental gradients to disperse throughout the vast expanse of the Amazon forest.

The presence of the American Dry Diagonal, a corridor of open, mainly savanna vegetation, which separates Amazonia from the Atlantic Forest did not determine any significant genetic differentiation between the disjunct *A. spadiceus* populations found in these distinct biomes (Figure 1b), as observed in other bird species (Bocalini et al., 2023; Lavinia et al., 2015; Weir & Price, 2011). The formation of a single genetic group in the Cis‐Andean lineage may be related to the facilitation of dispersal across the different corridors formed between these biomes during the different climatic phases of the Pleistocene (Batalha‐Filho et al., 2013; Nascimento et al., 2021), which would be consistent with the findings of Mendonça et al. (2022). One of these corridors would have been a route further to the north, along the northern coast of Brazil, in the states of Maranhão, Piauí, Ceará, and Rio Grande do Norte (Batalha‐Filho et al., 2013), formed by isolated humid forest refugia within the semi‐arid Caatinga dry forest (Andrade‐Lima, 1981). In fact, *A. spadiceus* has been recorded in this area (Girão et al., 2007). These cloud forest isolates may have been important links for disjunct species in the Amazonian and Atlantic forests during the Plio‐Pleistocene (Batalha‐Filho et al., 2013). But it is important to note that these findings are based on data on only two specimens from the Atlantic Forest, which do not contemplate the full complexity of the distribution of the species in this region. In this context, we would emphasize that the present study was the first multilocus analysis of the subspecies in this environment, and that further studies will be required, covering a more ample sample and additional genomic data, to provide a more robust and conclusive interpretation of the evolutionary history of the lineage in this region.

### 4.3 Demographic history

The final glaciation of the Pleistocene lasted from approximately 126,000 to 11,700 years ago (Corrêa, 2021; ICS, 2023), a period that favored a highly dynamic scenario in many of the areas of montane forest in Central America (Ornelas et al., 2013; Piperno et al., 2007; Still et al., 1999). These forests would have migrated latitudinally, both north‐ and southward, driven by climatic fluctuations (Ornelas et al., 2013; Piperno et al., 2007), which may have provided frequent opportunities for connectivity between the taxa (Barber & Klicka, 2010). We detected a discreet trend of demographic expansion in the Trans‐Andean lineage, which occurred in the middle of the final glaciation, at around 50,000 years ago, when these migrations would have taken place (Figure 4a), which is supported by the climatic scenario of 21,000 years ago, reflecting the amplification of the zones of niche suitability (see Appendix S5 in Supporting Information, Figure S1). This lineage may have been influenced by changes in the distribution of vegetation cover in the region. This is supported by evidence from climate models (Still et al., 1999) and paleoecological studies (Piperno et al., 2007), indicating that these forests expanded during this period, impacting the region's fauna.

The marked increase in the size of the population of the Cis‐Andean lineage (Figure 4b) occurred during the second phase of the last glaciation, between 75,000 and 30,000 years ago. This was a period of increasingly intense and prolonged cold, which peaked during the LGM, 23,000–19,000 years ago (Corrêa, 2021). As for the Trans‐Andean lineage, the climate zones were highly suitable for the Cis‐Andean lineage, which is consistent with our proposal of population expansion. These more intense cold periods impacted the role of rivers as barriers, in particular in the Amazon basin, favoring the distribution of some bird groups (Ribas et al., 2011). The environmental changes that occurred during this period influenced the population dynamics of the lineage. This scenario is consistent with the evidence of recent population expansion in other groups, as observed in the genus *Rhegmatorhina*, as a result of the changes in the drainage patterns of the Amazonian rivers and alterations in the forest cover during the Pleistocene (Ribas et al., 2018). This way, the expansion of the lineages of *A. spadiceus* may have been potentialized as a result of the changes in forest cover provoked by the fluctuations in climate.

The observed phylogeographic profile of *A. spadiceus* provides interesting insights into the drivers of diversification in the Neotropics, emphasizing the role of corridors north of the Andes. In addition to contrasting classic examples described for Neotropical avifauna in response to major biogeographic barriers, it expands our understanding of avian biodiversity formation in this part of the globe.

## AUTHOR CONTRIBUTIONS

**Patrícia Mendonça:** Conceptualization (lead); data curation (lead); formal analysis (lead); investigation (lead); methodology (lead); resources (supporting); software (lead); supervision (supporting); visualization (lead); writing – original draft (lead); writing – review and editing (lead). **Lincoln Silva Carneiro:** Conceptualization (supporting); formal analysis (supporting); funding acquisition (equal); methodology (supporting); resources (supporting); writing – original draft (supporting). **Victor Leandro‐Silva:** Data curation (supporting); formal analysis (supporting); methodology (supporting); software (supporting); writing – review and editing (supporting). **Juliana Araripe:** Formal analysis (supporting); funding acquisition (supporting); investigation (supporting); resources (supporting); writing – original draft (supporting); writing – review and editing (supporting). **Alexandre Aleixo:** Funding acquisition (equal); project administration (equal). **Péricles S. Rêgo:** Conceptualization (lead); formal analysis (lead); funding acquisition (lead); investigation (equal); methodology (supporting); project administration (lead); resources (equal); supervision (lead); visualization (equal); writing – original draft (lead); writing – review and editing (lead).

## FUNDING INFORMATION

The authors are grateful to the Brazilian National Council for Scientific and Technological Development, CNPq (National Council for Scientific and Technological Development): JA – processes 312404/2019‐0 and 439040/2018‐3, and PSR – process 311539/2019‐0, and the Coordination for Higher Education Personnel Training, CAPES (Coordination for the Improvement of Higher Education Personnel: PM – process 88887.649545/2021‐00) for supporting this research. A is supported by a Productivity Research Fellowship from the Brazilian National and Technological Development Council – CNPq (#309243/2023‐8).

## CONFLICT OF INTEREST STATEMENT

There are no conflicts of interest, and all authors are aware of the submission of the information disclosed in this work.

## Supporting information


Appendix S1.

Appendix S2.

## References

[ece370331-bib-0001] Alfaro, J. W. L. , Boubli, J. P. , Paim, F. P. , Ribas, C. C. , Silva, M. N. F. , Messias, M. R. , Röhe, F. , Mercês, M. P. , Júnior, J. S. S. , Silva, C. R. , Pinho, G. M. , Koshkarian, G. , Nguyen, M. T. , Harada, M. L. , Rabelo, F. M. , Queiroz, H. L. , Alfaro, M. E. , & Farias, I. P. (2015). Biogeography of squirrel monkeys (genus *Saimiri*): South‐central Amazon origin and rapid pan‐Amazonian diversification of a lowland primate. Molecular Phylogenetics and Evolution, 82, 436–454. 10.1016/j.ympev.2014.09.004 25305518

[ece370331-bib-0002] Allouche, O. , Tsoar, A. , & Kadmon, R. (2006). Assessing the accuracy of species distribution models: Prevalence, kappa and the true skill statistic (TSS). Journal of Applied Ecology, 43(6), 1223–1232. 10.1111/j.1365-2664.2006.01214.x

[ece370331-bib-0095] Andrade‐Lima, D. (1981). The caatingas dominium. Revista Brasileira de Botânica, 4, 149–163.

[ece370331-bib-0003] Anunciação, P. R. , Ernst, R. , Martello, F. , Vancine, M. H. , Carvalho, L. M. T. , & Ribeiro, M. C. (2023). Climate‐driven loss of taxonomic and functional richness in Brazilian Atlantic Forest anurans. Perspectives in Ecology and Conservation, 21(4), 274–285. 10.1016/j.pecon.2023.09.001

[ece370331-bib-0004] Araújo, M. B. , & New, M. (2007). Ensemble forecasting of species distribution. Trends in Ecology & Evolution, 22(1), 42–47. 10.1016/j.tree.2006.09.010 17011070

[ece370331-bib-0093] Bagley, J. C. , & Johnson, J. B. (2014). Phylogeography and biogeography of the lower Central American Neotropics: Diversification between two continents and between two seas. Biological Reviews, 89(4), 767–790. 10.1111/brv.12076 24495219

[ece370331-bib-0005] Barber, B. R. , & Klicka, J. (2010). Two pulses of diversification across the isthmus of Tehuantepec in a montane Mexican bird fauna. Proceedings of the Royal Society B: Biological Sciences, 277(1694), 2675–2681. 10.1098/rspb.2010.0343 PMC2982039 20410037

[ece370331-bib-0006] Barrier, E. , Velasquillo, L. , Chavez, M. , & Gaulon, R. (1998). Neotectonic evolution of the isthmus of Tehuantepec (southeastern Mexico). Tectonophysics, 287(1–4), 77–96.

[ece370331-bib-0007] Barry, S. , & Elith, J. (2006). Error and uncertainty in habitat models. Journal of Applied Ecology, 43(3), 413–423. 10.1111/j.1365-2664.2006.01136.x

[ece370331-bib-0008] Batalha‐Filho, H. , Fjeldså, J. , Fabre, P. H. , & Miyaki, C. Y. (2013). Connections between the Atlantic and the Amazonian Forest avifaunas represent distinct historical events. Journal of Ornithology, 154(1), 41–50. 10.1007/s10336-012-0866-7

[ece370331-bib-0009] Berv, J. S. , Campagna, L. , Feo, T. J. , Castro‐Astor, I. , Ribas, C. C. , Prum, R. O. , & Lovette, I. J. (2021). Genomic phylogeography of the White crowned Manakin (*Pseudopipra pipra*; Aves: Pipridae) illuminates a continental‐scale radiation out of the Andes. Molecular Phylogenetics and Evolution, 164, 107205. 10.1016/j.ympev.2021.107205 34015448

[ece370331-bib-0010] Bocalini, F. , Bolívar‐Leguizamón, S. D. , Silveira, L. F. , & Bravo, G. A. (2023). Amazonian colonization from the Atlantic Forest: New perspectives on the connections of south American tropical forests. Molecular Ecology, 32(24), 6874–6895. 10.1111/mec.17180 37902123

[ece370331-bib-0012] Broennimann, O. , Di Cola, V. , & Guisan, A. (2023). *Ecospat: Spatial ecology miscellaneous methods* (R package version 4.0.0). https://CRAN.R‐project.org/package=ecospat

[ece370331-bib-0013] Brown, J. L. (2014). SDMtoolbox: A python‐based GIS toolkit for landscape genetic, biogeographic and species distribution model analyses. Methods in Ecology and Evolution, 5(7), 694–700. 10.1111/2041-210X.12200 PMC5721907 29230356

[ece370331-bib-0014] Brown, J. L. , Hill, D. J. , Dolan, A. M. , & Haywood, A. M. (2018). PaleoClim, high spatial resolution paleoclimate surfaces for global land areas. Scientific Data, 5, 180254. 10.1038/sdata.2018.254 30422125 PMC6233254

[ece370331-bib-0015] Burney, C. W. , & Brumfield, R. T. (2009). Ecology predicts levels of genetic differentiation in Neotropical birds. The American Naturalist, 174(3), 358–368. 10.1086/603613 19627230

[ece370331-bib-0016] Carpenter, G. , Gillison, A. N. , & Winter, J. (1993). DOMAIN: A flexible modeling procedure for mapping potential distribution of plants and animals. Biodiversity and Conservation, 2(6), 667–680.

[ece370331-bib-0017] Castillo‐Chora, V. J. , Zamudio‐Beltran, L. , Pozo, C. , & Hernandez‐Banos, B. E. (2021). Phylogeography of *Habia fuscicauda* (Cardinalidae) indicates population isolation, genetic divergence, and demographic changes during the quaternary climate shifts in the Mesoamerican rainforest. Journal of Ornithology, 162, 961–976. 10.1007/s10336-021-01904-x

[ece370331-bib-0018] Coates, A. G. , & Stallard, F. R. (2013). How old is the isthmus of Panama? Marine Sciences, 89, 801–813. 10.5343/bms.2012.1076

[ece370331-bib-0019] Corrêa, I. C. S. (2021). Variações climáticas no Quaternário. ISBN: 978‐65‐00‐21570‐0.

[ece370331-bib-0020] D'Horta, F. M. , Cuervo, A. M. , Ribas, C. C. , Brumfield, R. T. , & Miyaki, C. Y. (2013). Phylogeny and comparative phylogeography of *Sclerurus* (Aves: Furnariidae) reveal constant and cryptic diversification in an old radiation of rain forest understorey specialists. Journal of Biogeography, 40(1), 37–49. 10.1111/j.1365-2699.2012.02760.x

[ece370331-bib-0021] Diniz‐Filho, J. A. F. , Bini, L. M. , Rangel, T. F. , Loyola, R. D. , Hof, C. , Nogués‐Bravo, D. , & Araújo, M. B. (2009). Partitioning and mapping uncertainties in ensembles of forecasts of species turnover under climate change. Ecography, 32(6), 897–906. 10.1111/j.1600-0587.2009.06196.x

[ece370331-bib-0023] Drummond, A. J. , Suchard, M. A. , Xie, D. , & Rambaut, A. (2012). Bayesian phylogenetics‐with BEAUti and the BEAST 1.7. Molecular Biology and Evolution, 29(8), 1969–1973. 10.1093/molbev/mss075 22367748 PMC3408070

[ece370331-bib-0025] Felsenstein, J. (2004). PHYLIP (phylogeny inference package) version 3.6. Distributed by the author. http://www.evolution.gs.washington.edu/phylip.html

[ece370331-bib-0026] Fernandes, A. M. , Wink, M. , Sardelli, C. H. , & Aleixo, A. (2014). Multiple speciation across the Andes and throughout Amazonia: The case of the spot‐backed antbird species complex (*Hylophylax naevius*/*Hylophylax naevioides*). Journal of Biogeography, 41(6), 1094–1104. 10.1111/jbi.12277

[ece370331-bib-0028] Franklin, J. (2009). Mapping species distributions: Spatial inference and predictions. Cambridge University Press.

[ece370331-bib-0029] Fu, Y. X. (1997). Statistical test of neutrality of mutations against population growth, hitchhiking and background selection. Genetics, 147(2), 915–925. 10.1093/genetics/147.2.915 9335623 PMC1208208

[ece370331-bib-0030] GBIF.org . (2024). GBIF occurrence download. 10.15468/dl.jqyh6g

[ece370331-bib-0032] Girão, W. , Albano, C. , Pinto, T. , & Silveira, L. F. (2007). Avifauna da Serra de Baturité: dos naturalistas à atualidade. In T. S. Oliveira & F. S. Araújo (Eds.), Biodiversidade e conservação da biota na Serra de Baturité, Ceará. Edições UFC.

[ece370331-bib-0033] Gregory‐Wodzicki, K. M. (2000). Uplift history of the central and northern Andes: A review. Geological Society of America Bulletin, 112(7), 1091–1105. 10.1130/0016-7606(2000)112<1091>2.0.CO;2

[ece370331-bib-0034] Guisan, A. , Edwards, T. C., Jr. , & Hastie, T. (2002). Generalized linear and generalized additive models in studies of species distributions: Setting the scene. Ecological Modelling, 157(2–3), 89–100. 10.1016/S0304-3800(02)00204-1

[ece370331-bib-0036] Haffer, J. (1967). Speciation in Colombian forest birds west of the Andes. American Museum Novitates, 2294, 1–57. http://hdl.handle.net/2246/3087

[ece370331-bib-0037] Hall, T. A. (1999). BIOEDIT: A user‐friendly biological sequence alignment editor and analysis program for windows 95/98/NT. Nucleic Acids Symposium, 41, 95–98.

[ece370331-bib-0038] Harrigan, R. J. , Mazza, M. E. , & Sorenson, M. D. (2008). Computation versus cloning: Evaluation of two methods for haplotype determination. Molecular Ecology, 17, 1239–1248. 10.1111/j.1755-0998.2008.02241.x 21586011

[ece370331-bib-0040] Heled, J. , & Drummond, A. J. (2008). Bayesian inference of population size history from multiple loci. BMC Evolutionary Biology, 8, 1–15. https://bit.ly/2Oec7M8 18947398 10.1186/1471-2148-8-289 PMC2636790

[ece370331-bib-0041] Hijmans, R. J. , Phillips, S. , Leathwick, J. , & Elith, J. (2022). Dismo: Species distribution modeling. R package version 1.3‐9. https://CRAN.R‐project.org/package=dismo

[ece370331-bib-0042] Hoorn, C. , Wesselingh, F. P. , Ter Steege, H. , Bermudez, M. A. , Mora, A. , Sevink, J. , Sanmartín, I. , Sanchez‐Meseguer, A. , Anderson, C. L. , Figueiredo, J. P. , Jaramillo, C. , Riff, D. , Negri, F. R. , Hooghiemstra, H. , Lundberg, J. , Stadler, T. , Särkinen, T. , & Antonelli, A. (2010). Amazonia through time: Andean uplift, climate change, landscape evolution, and biodiversity. Science, 330(6006), 927–931. 10.1126/science.1194585 21071659

[ece370331-bib-0043] International Commission on Stratigraphy (ICS) . (2023). International Chronostratigraphic Chart. https://stratigraphy.org/

[ece370331-bib-0044] Jiménez‐Valverde, A. , Peterson, A. T. , Soberón, J. , Overton, J. M. , Aragón, P. , & Lobo, J. M. (2011). Use of niche models in invasive species risk assessments. Biological Invasions, 13, 2785–2797. 10.1007/s10530-011-9963-4

[ece370331-bib-0045] Johnson, O. , Ribas, C. C. , Aleixo, A. , Naka, L. N. , Harvey, M. G. , & Brumfield, R. T. (2023). Amazonian birds in more dynamic habitats have less population genetic structure and higher gene flow. Molecular Ecology, 32(9), 2186–2205. 10.1111/mec.16886 36798996

[ece370331-bib-0046] Karatzoglou, A. , Smola, A. , Hornik, K. , & Seileis, A. (2004). kernlab—an S4 package for kernel methods in R. Journal of Statistical Software, 11(9), 1–20. 10.18637/jss.v011.i09

[ece370331-bib-0047] Kumar, S. , Stecher, G. , Li, M. , Knyaz, C. , & Tamura, K. (2018). MEGA X: Molecular evolutionary genetics analysis across computing platforms. Molecular Biology and Evolution, 35(6), 1547–1549. 10.1093/molbev/msy096 29722887 PMC5967553

[ece370331-bib-0048] Lanfear, R. , Frandsen, P. B. , Wright, A. M. , Senfeld, T. , & Calcott, B. (2016). PartitionFinder 2: New methods for selecting partitioned models of evolution for molecular and morphological phylogenetic analyses. Molecular Biology and Evolution, 34(3), 772–773. 10.1093/molbev/msw260 28013191

[ece370331-bib-0049] Lavinia, P. D. , Escalante, P. , García, N. C. , Barreira, A. S. , Trujillo‐Arias, N. , Tubaro, P. L. , Naoki, K. , Miyaki, C. Y. , Santos, F. R. , & Lijtmaer, D. A. (2015). Continental‐scale analysis reveals deep diversification within the polytypic red‐crowned ant tanager (*Habia rubica*; Cardinalidae). Molecular Phylogenetics and Evolution, 89, 182–193. 10.1016/j.ympev.2015.04.018 25929787

[ece370331-bib-0050] Liaw, A. , & Wiener, M. (2002). Classification and regression by randomForest. R News, 2(3), 18–22.

[ece370331-bib-0051] Librado, P. , & Rozas, J. (2009). DnaSP v5.10: A software for comprehensive analysis of DNA polymorphism data. Bioinformatics, 25(11), 1451–1452. 10.1093/bioinformatics/btp187 19346325

[ece370331-bib-0052] Liu, C. , Newell, G. , & White, M. (2016). On the selection of thresholds for predicting species occurrence with presence‐only data. Ecology and Evolution, 6, 337–348. 10.1002/ece3.1878 26811797 PMC4716501

[ece370331-bib-0053] Luna, L. W. , Dias, C. , Pichorim, M. , Leandro‐Silva, V. , Biancalana, R. N. , de Girão e Silva, W. A. , Araripe, J. , & Sena do Rêgo, P. (2022). Historical climate change as driver of populational range expansion and differentiation in a rare and partially migratory Neotropical bird. Journal of Ornithology, 163(1), 495–507. 10.1007/s10336-022-01846-2

[ece370331-bib-0054] Marshall, C. , & Liebherr, J. (2000). Cladistic biogeography of the Mexican transition zone. Journal of Biogeography, 27(1), 203–216. 10.1046/j.1365-2699.2000.00388.x

[ece370331-bib-0055] Mendonça, P. , Dias, C. , Aleixo, A. , Carneiro, L. S. , Araripe, J. , & Rêgo, P. S. (2022). Diversification across the isthmus of Tehuantepec explains the phylogeographic arrangement of the widespread bright‐rumped Attila (*Attila spadiceus*; Tyrannidae) and reveals the existence of two major lineages. Journal of Ornithology, 163(1), 327–332. 10.1007/s10336-021-01928-3

[ece370331-bib-0091] Miller, M. A. , Pfeiffer, W. , & Schwartz, T. (2010). Creating the CIPRES Science Gateway for inference of large phylogenetic trees. In Proceedings of the gateway computing environments workshop (GCE) (pp. 1–8). IEEE, New Orleans, LA. 10.1109/GCE.2010.5676129

[ece370331-bib-0056] Nascimento, N. F. F. , Agne, C. E. Q. , Batalha‐Filho, H. , & Araújo, H. F. P. (2021). Population history of the blue‐backed Manakin (*Chiroxiphia pareola*) supports Plio‐Pleistocene diversification in the Amazon and shows a recent connection with the Forest. Journal of Ornithology, 162, 549–563. 10.1007/s10336-020-01845-x

[ece370331-bib-0057] Nix, H. A. (1986). A biogeographic analysis of Australian elapid snake. In R. Longmore (Ed.), Atlas of elapid snakes of Australia (pp. 4–15). Australian Government Publishing Service.

[ece370331-bib-0058] Ornelas, J. F. , Sosa, V. , Soltis, D. E. , Daza, J. M. , González, C. , Soltis, P. S. , Gutiérrez‐Rodríguez, C. , Monteros, A. E. , Castoe, T. A. , Bell, C. , & Ruiz‐Sanchez, E. (2013). Comparative phylogeographic analyses illustrate the complex evolutionary history of threatened cloud forests of northern Mesoamerica. PLoS One, 8, e56283. 10.1371/journal.pone.0056283 23409165 PMC3567015

[ece370331-bib-0059] Peres, E. A. , Sobral‐Souza, T. , Perez, M. F. , Bonatelli, I. A. , Silva, D. P. , Silva, M. J. , & Solferini, V. N. (2015). Pleistocene niche stability and lineage diversification in the subtropical spider *Araneus omnicolor* (Araneidae). PLoS One, 10, e0121543. 10.1371/journal.pone.0121543 25856149 PMC4391720

[ece370331-bib-0060] Peterson, A. T. , Soberón, J. , Pearson, R. G. , Anderson, R. P. , Martínez‐Meyer, E. , Nakamura, M. , & Araújo, M. B. (2011). Ecological niches and geographic distributions (MPB‐49). Princeton University Press.

[ece370331-bib-0061] Philips, S. J. , & Dudik, M. (2008). Modeling of species distributions with Maxent: New extensions and a comprehensive evaluation. Ecography, 31(2), 161–175. 10.1111/j.0906-7590.2008.5203.x

[ece370331-bib-0062] Piperno, D. R. , Moreno, J. E. , Iriarte, J. , Holst, I. , Lachniet, M. , Jones, J. G. , & Ranere, A. J. (2007). Late Pleistocene and Holocene environmental history of the Iguala Valley, central balsas watershed of Mexico. Proceedings of the National Academy of Sciences USA, 104(29), 11874–11881. 10.1073/pnas.0703442104 PMC1880864 17537917

[ece370331-bib-0092] Rambaut, A. , Drummond, A. J. , Xie, D. , Baele, G. , & Suchard, M. A. (2018). Tracer1.7.1‐analyse results from bayesian MCMC programs such as BEAST & MrBayes. Systematic Biology, 67(5), 901–904. 10.1093/sysbio/syy032 29718447 PMC6101584

[ece370331-bib-0064] Reis, C. A. , Dias, C. , Araripe, J. , Aleixo, A. , Anciães, M. , Sampaio, I. , Schneider, H. , & Rêgo, P. S. (2019). Multilocus data of a manakin species reveal cryptic diversification moulded by vicariance. Zoologica Scripta, 49(2), 1–16. 10.1111/zsc.12395

[ece370331-bib-0066] Ribas, C. C. , Moyle, R. G. , Miyaki, C. Y. , & Cracraft, J. (2007). The assembly of montane biotas: Linking Andean tectonics and climatic oscillations to independent regimes of diversification in Pionus parrots. Proceedings of the Royal Society of London B: Biological Sciences, 274(1620), 2399–2408. 10.1098/rspb.2007.0613 PMC2274971 17686731

[ece370331-bib-0065] Ribas, C. C. , Aleixo, A. , Nogueira, A. C. , Miyaki, C. Y. , & Cracraft, J. (2011). A palaeobiogeographic model for biotic diversification within Amazonia over the past three million years. Proceedings of the Royal Society of London B: Biological Sciences, 279(1729), 681–689. 10.1098/rspb.2011.1120 PMC3248724 21795268

[ece370331-bib-0096] Ribas, C. C. , Aleixo, A. , Nogueira, A. C. , Miyaki, C. Y. , & Cracraft, J. (2018). Amazonian diversification: Palaeogeography, landscape evolution, and species diversification. Journal of Biogeography, 45(5), 915–931. 10.1111/jbi.13169

[ece370331-bib-0067] Rocha‐Méndez, A. , Sánchez‐González, L. A. , Arbeláez‐Cortés, E. , & Navarro‐Sigüenza, A. G. (2018). Phylogeography indicates incomplete genetic divergence among phenotypically differentiated montane forest populations of *Atlapetes albinucha* (Aves, Passerellidae). ZooKeys, 809, 125–148. 10.3897/zookeys.809.28743 PMC6306474 30598618

[ece370331-bib-0068] Rodríguez‐Gómez, F. , & Ornelas, J. F. (2018). Genetic structuring and secondary contact in the white‐chested *Amazilia* hummingbird species complex. Journal of Biogeography, 49(1), 1–19. 10.1111/jav.01536

[ece370331-bib-0069] Ronquist, F. , Teslenko, M. , Van der Mark, P. , Ayres, D. , Darling, A. , Höhna, S. , Larget, B. , Liu, L. , Suchard, M. A. , & Huelsenbeck, J. P. (2012). MrBayes 3.2: Efficient Bayesian phylogenetic inference and model choice across a large model space. Systematic Biology, 61(3), 539–542. 10.1093/sysbio/sys029 22357727 PMC3329765

[ece370331-bib-0070] Salzburger, W. , Ewing, G. B. , & Von Haeseler, A. (2011). The performance of phylogenetic algorithms in estimating haplotype genealogies with migration. Molecular Ecology, 20(9), 1952–1963. 10.1111/j.1365-294X.2011.05066.x 21457168

[ece370331-bib-0097] Sanger, F. , Nicklen, S. , & Coulson, A. R. (1977). DNA sequencing with chain‐terminating inhibitors. Proceedings of the National Academy of Sciences of the United States of America, 74(12), 5463–5467. https://bit.ly/2xUoQJ3 271968 10.1073/pnas.74.12.5463 PMC431765

[ece370331-bib-0071] Schoener, T. W. (1968). Anolis lizards of Bimini: Resource partitioning in a complex fauna. Ecology, 49(4), 704–726. 10.2307/1935534

[ece370331-bib-0073] Silva, S. M. , Peterson, A. T. , Carneiro, L. , Burlamaqui, T. C. T. , Ribas, C. C. , Sousa‐Neves, T. , Miranda, L. S. , Fernandes, A. M. , D'Horta, F. M. , Araújo‐Silva, L. E. , Batista, R. , Bandeira, C. H. M. M. , Dantas, S. M. , Ferreira, M. , Martins, D. M. , Oliveira, J. , Rocha, T. C. , Sardelii, C. H. , Thom, G. , … Aleixo, A. (2019). A dynamic continental moisture gradient drove Amazonian bird diversification. Science Advances, 5(7), 1–10. 10.1126/sciadv.aat5752 PMC6609164 31281878

[ece370331-bib-0074] Smith, B. T. , & Klicka, J. (2010). The profound influence of the late Pliocene Panamanian uplift on the exchange, diversification, and distribution of New World birds. Ecography, 33(2), 333–342. 10.1111/j.1600-0587.2009.06335.x

[ece370331-bib-0075] Smith, B. T. , McCormack, J. E. , Cuervo, A. M. , Hickerson, M. J. , Aleixo, A. , Cadena, C. D. , Pérez‐Emán, J. , Burney, C. W. , Xie, X. , Harvey, N. G. , Faircloth, B. C. , Glenn, T. C. , Derryberry, E. P. , Prejean, J. , Fields, S. , & Harvey, M. G. (2014). The drivers of tropical speciation. Nature, 515(7527), 406–409. https://go.nature.com/2GbGMUK 25209666 10.1038/nature13687

[ece370331-bib-0076] Sobral‐Souza, T. , Lima‐Ribeiro, M. S. , & Solferini, V. N. (2015). Biogeography of Neotropical rainforests: Past connections between Amazon and Atlantic Forest detected by ecological niche modeling. Evolutionary Ecology, 29, 643–655. 10.1007/s10682-015-9775-8

[ece370331-bib-0078] Stephens, M. , & Donnelly, P. A. (2003). Comparison of Bayesian methods for haplotype reconstruction from population genotype data. American Journal of Human Genetics, 73, 1162–1169. 10.1086/379378 14574645 PMC1180495

[ece370331-bib-0079] Still, C. J. , Foster, P. N. , & Schneider, S. H. (1999). Simulating the effects of climate change on tropical montane cloud forests. Nature, 398, 608–610. 10.1038/19255

[ece370331-bib-0080] Tajima, F. (1989). Statistical method for testing the neutral mutation hypothesis by DNA polymorphism. Genetics, 123(3), 585–595. 10.1093/genetics/123.3.585 2513255 PMC1203831

[ece370331-bib-0081] Tax, D. M. J. , & Duin, R. P. W. (2004). Support vector data description. Machine Learning, 54, 45–66. 10.1023/B.0000008082.16305.43

[ece370331-bib-0082] Thompson, J. D. , Gibson, T. J. , Plewniak, F. , Jeanmougin, F. , & Higgins, D. G. (1997). The CLUSTAL_X windows interface: Flexible strategies for multiple sequence alignment aided by quality analysis tools. Nucleic Acids Research, 25(24), 4876–4882. 10.1093/nar/25.24.4876 9396791 PMC147148

[ece370331-bib-0083] Thuiller, W. , Brotons, L. , Araujo, M. B. , & Lavorel, S. (2004). Effects of restricting environmental range of data to project current and future species distributions. Ecography, 27(2), 165–172. https://www.jstor.org/stable/3683828

[ece370331-bib-0084] Vaidya, G. , Lohman, D. J. , & Meir, R. (2001). SequenceMatrix: Concatenation software for the fast assembly of multi‐gene datasets with maximum sequence flexibility. Molecular Ecology Resources, 11, 342–355. 10.1111/17550998.12196

[ece370331-bib-0085] Wallace, A. R. (1852). On the monkeys of the Amazon. Proceedings of the Zoological Society of London, 20, 107–110.

[ece370331-bib-0086] Walther, B. (2020). Bright‐rumped Attila (*Attila spadiceus*), versão 1.0. In J. Hoyo , A. Elliott , J. Sargatal , D. A. Christie , & E. Juana (Eds.), Birds of the world. Cornell Lab of Ornithology. 10.2173/bow.brratt1.01

[ece370331-bib-0087] Warren, D. L. , Glor, R. E. , & Turelli, M. (2008). Environmental niche equivalency versus conservatism: Quantitative approaches to niche evolution. Evolution, 62(11), 2868–2883. 10.1111/j.1558-5646.2008.00482.x 18752605

[ece370331-bib-0088] Weir, J. T. , & Price, M. (2011). Andean uplift promotes lowland speciation through vicariance and dispersal in *Dendrocincla* woodcreepers. Molecular Ecology, 20(21), 4550–4563. 10.1111/j.1365-294X.2011.05294.x 21981112

[ece370331-bib-0089] Weir, J. T. , & Schluter, D. (2008). Calibrating the avian molecular clock. Molecular Ecology, 17(10), 2321–2328. 10.1111/j.1365-294X.2008.03742.x 18422932

[ece370331-bib-0094] Yamaguchi, T. (2022). The populations of species ecologically adapted for dispersal may not be structured by geographic barriers: Insights from Amazon forest dispersal. Journal of Biogeography, 49(2), 345–356. 10.1111/1440-1703.12313

