# Supplementary material for: The influence of the forest corridors to the north of the Andes on the diversification of the bright‐rumped Attila, Attila spadiceus (Passeriformes, Tyrannidae), during the climatic oscillations of the middle Pleistocene
Source: Ecol Evol. 2025 Jan 21;15(1):e70331. doi: 10.1002/ece3.70331 (PMC11751253; doi:10.1002/ece3.70331)

**SUPPORTING INFORMATION**

The influence of the forest corridors to the north of the Andes on the diversification of the Bright-rumped Attila, *Attila spadiceus* (Passeriformes, Tyrannidae), during the climatic oscillations of the middle Pleistocene

Patrícia Mendonça, Lincoln Silva Carneiro, Victor Leandro-Silva, Alexandre Aleixo, Juliana Araripe, Péricles S. Rêgo

**APPENDIX 2. Table S2.** Genes used in the present study, primer combinations, and their sequences.

| **Gene** | **Primer** | **Sequence** | **Anelamento** | **Reference** |
| --- | --- | --- | --- | --- |
| CytB | 14996L  16064H | 5’ AGCCCCATCCAACATCTCTGCTTG 3’  5’ CTTCGATCTTTGGCTTACAAGAAC3’ | 48 ºC | Sorenson et al., 1999  Sorenson et al., 1999 |
| ND2 | L5216  H6313 | 5’ GGCCCATACCCGRAAAT 3’  5’ CTCTTATTTAAGGCTTTGAAGGC 3’ | 55 ºC | Hackett, 1996  Hackett, 1996 |
| I7BF | LI7BF  HI7BF | 5’ TC CCCAGTAGTATCTGCCATTAGGGTT 3’  5’ GGAGAAAACAGGACAATGACAATTCAC 3’ | 50 ºC | Prychitko & Moore, 1997  Prychitko & Moore, 1997 |
| I5BF | FIB5  FIB6 | 5’ CGCCATACAGAGTATACTGTGACA 3’  5’ GGCATCCTGGCGATTCTGAA 3’ | 53,5 ºC | Driskell & Christides, 2004  Driskell & Christides, 2004 |
| G3PDH | G3PDH13b  G3PDH14b | 5’ TCCACCTTTGATGCGGGTGCTGGCAT 3’  5’ AAGTCCACAACACGGTTGCTGTA 3’ | 60 ºC | Fjeldsa et al., 2003  Fjeldsa et al., 2003 |

**APPENDIX 3. Table S3.** Partitions and evolutionary models estimated in PartitionFinder.

| **Multilocus database to Bayesian Inference** |  | |
| --- | --- | --- |
| **Partition** | | **Model** |
| I7BF, I5BF, G3PDH (Position 1); ND2, CytB (Position 1 and 2) | | GTR+I+G |
| CytB, ND2 (Position 3) | | GTR+G |
| **Mitochondrial database to Bayesian Inference** | | |
| **Partition** | | **Model** |
| CytB (Position 1) | | K80 |
| ND2 (Position 1), ND2 (Position 2), CytB (Position 2), | | HKY+I |
| CytB (Position 3), ND2 (Position 3) | | GTR+G |
| **Multilocus database to tree species** | | **Model** |
| CytB, ND2 | | HKY+I+G |
| G3PDH, I5BF and I7BF | | GTR+I+G |
| **Multilocus database to EBSP - Trans-Andean lineage** | | **Model** |
| ND2, CytB, G3PDH | | HKY+I + G |
| I7BF, I5BF | | HKY+I |
| **Multilocus database to EBSP - Cis-Andean lineage** | | **Model** |
| ND2, CytB, G3PDH | | HKY+I |
| I7BF, I5B | | TRN+I |

**APPENDIX 4. Table S4.** Variables used to build the climatic models. Variables with correlation R > 0.70 were excluded.

| **Variable** | **Description** |
| --- | --- |
| Bio02 | Variation of the daytime average (average per month (temp Max - temp min)) |
| Bio05 | Max Temperature of Warmest Month |
| Bio07 | Temperature Annual Range (BIO5-BIO6) |
| Bio10 | Mean Temperature of Warmest Quarter |
| Bio12 | Annual Precipitation |
| Bio13 | Precipitation of the rainiest month. |
| Bio18 | Precipitation in the hottest quarter |

**APPENDIX 5. Figure S1.** Niche suitability model of trans- and cis-andine lineages in nine different temporal climate scenarios from a-c (see legend). Areas in red indicate high climate suitability, while areas tending towards blue show low or no climate suitability.


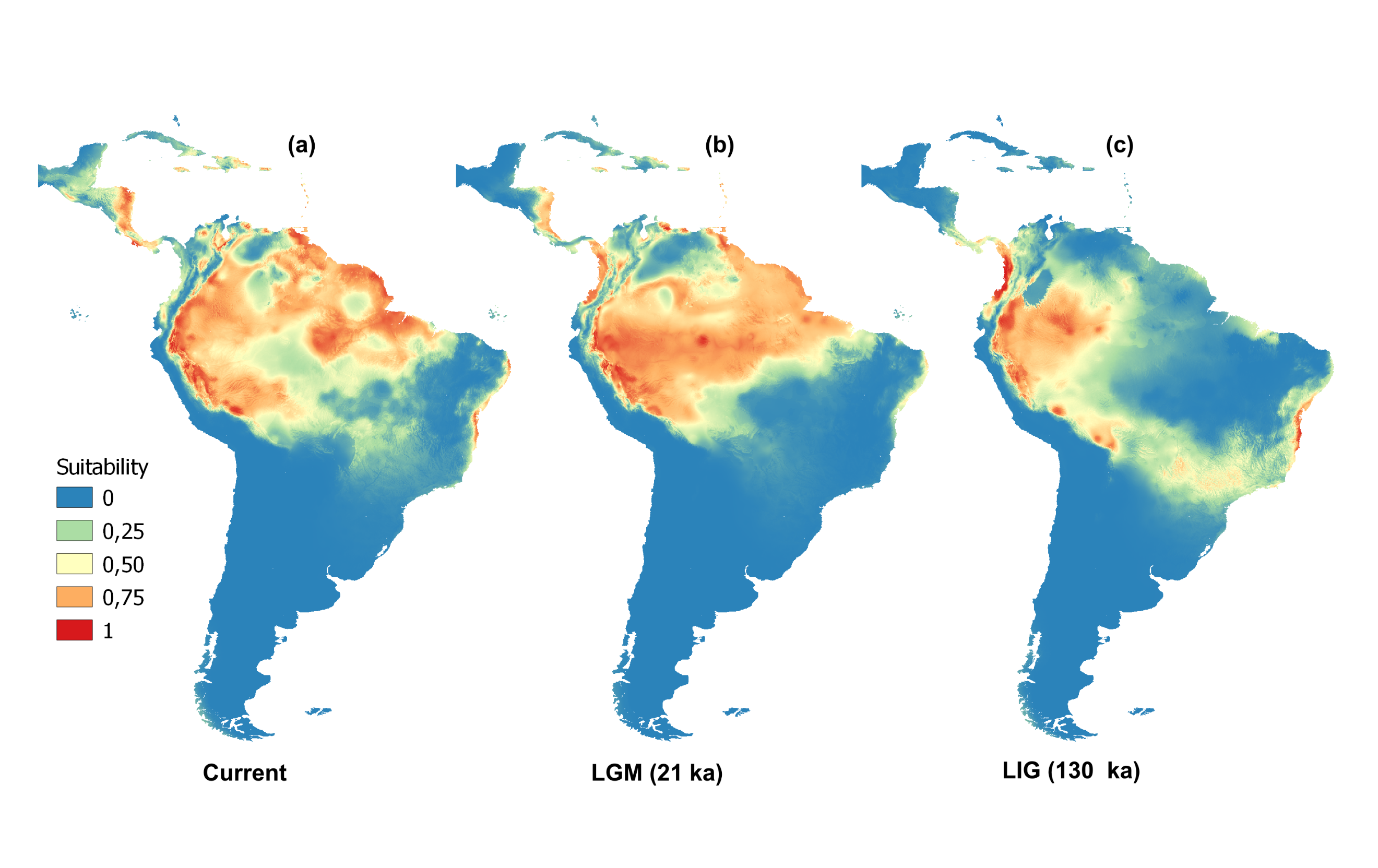

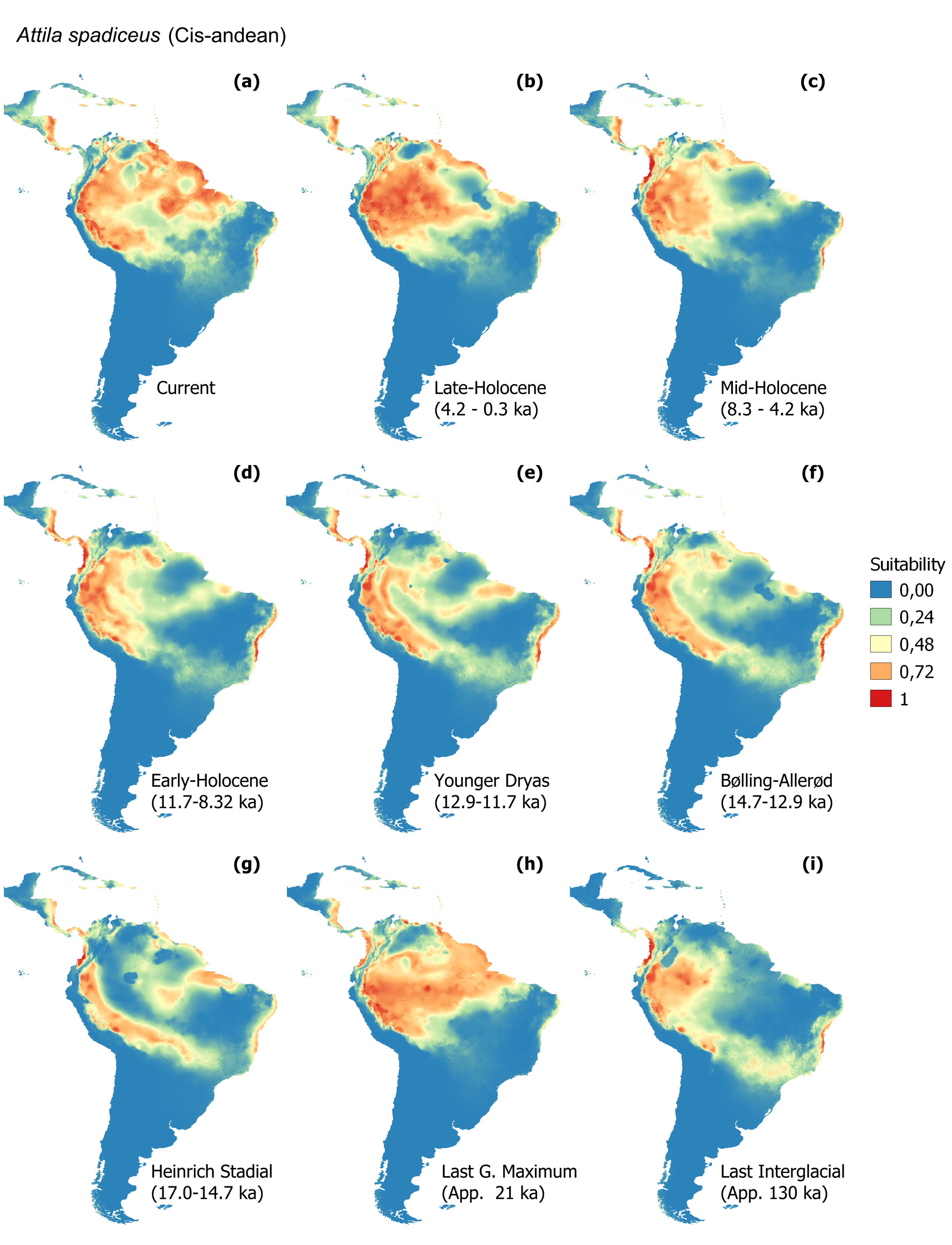

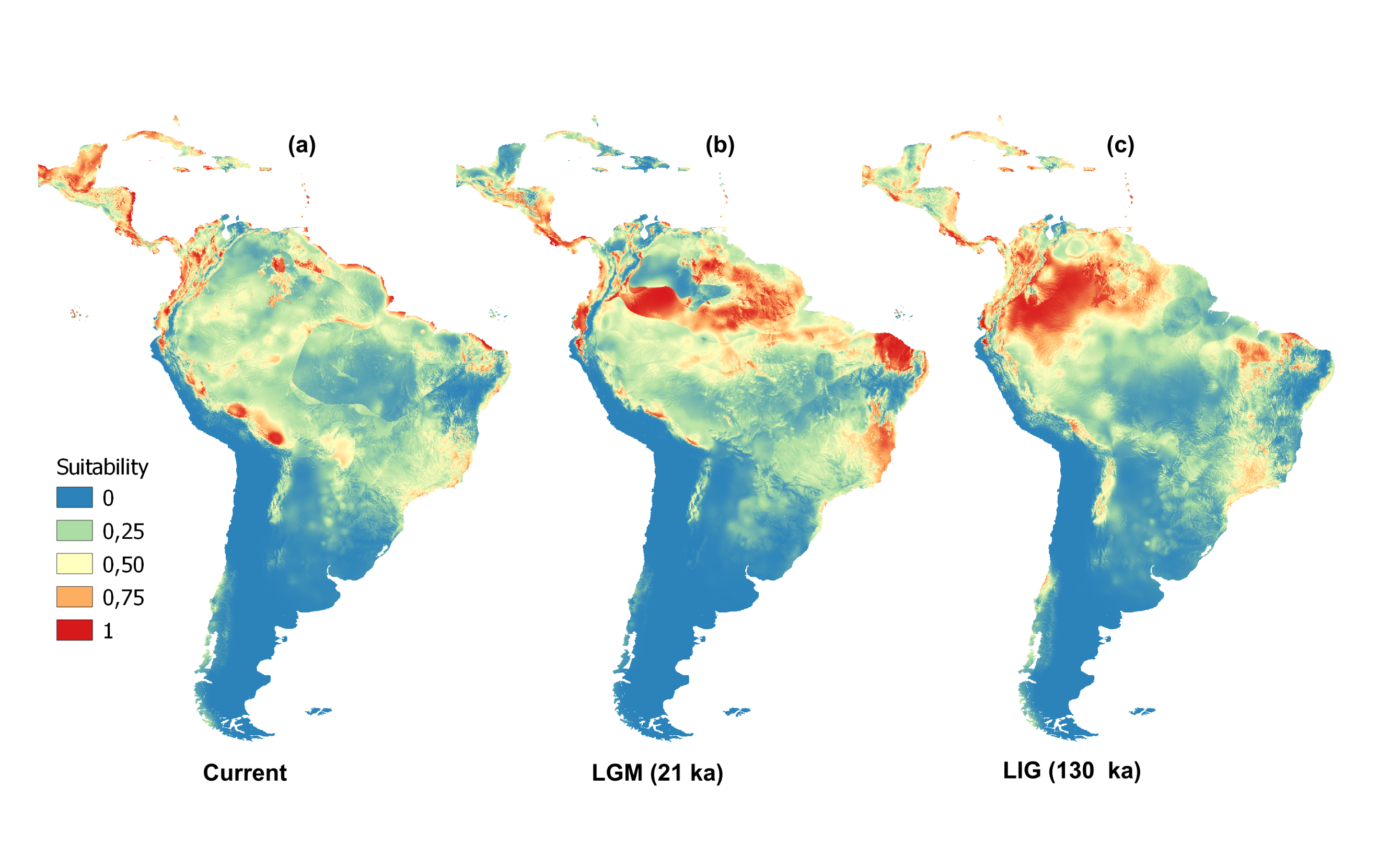

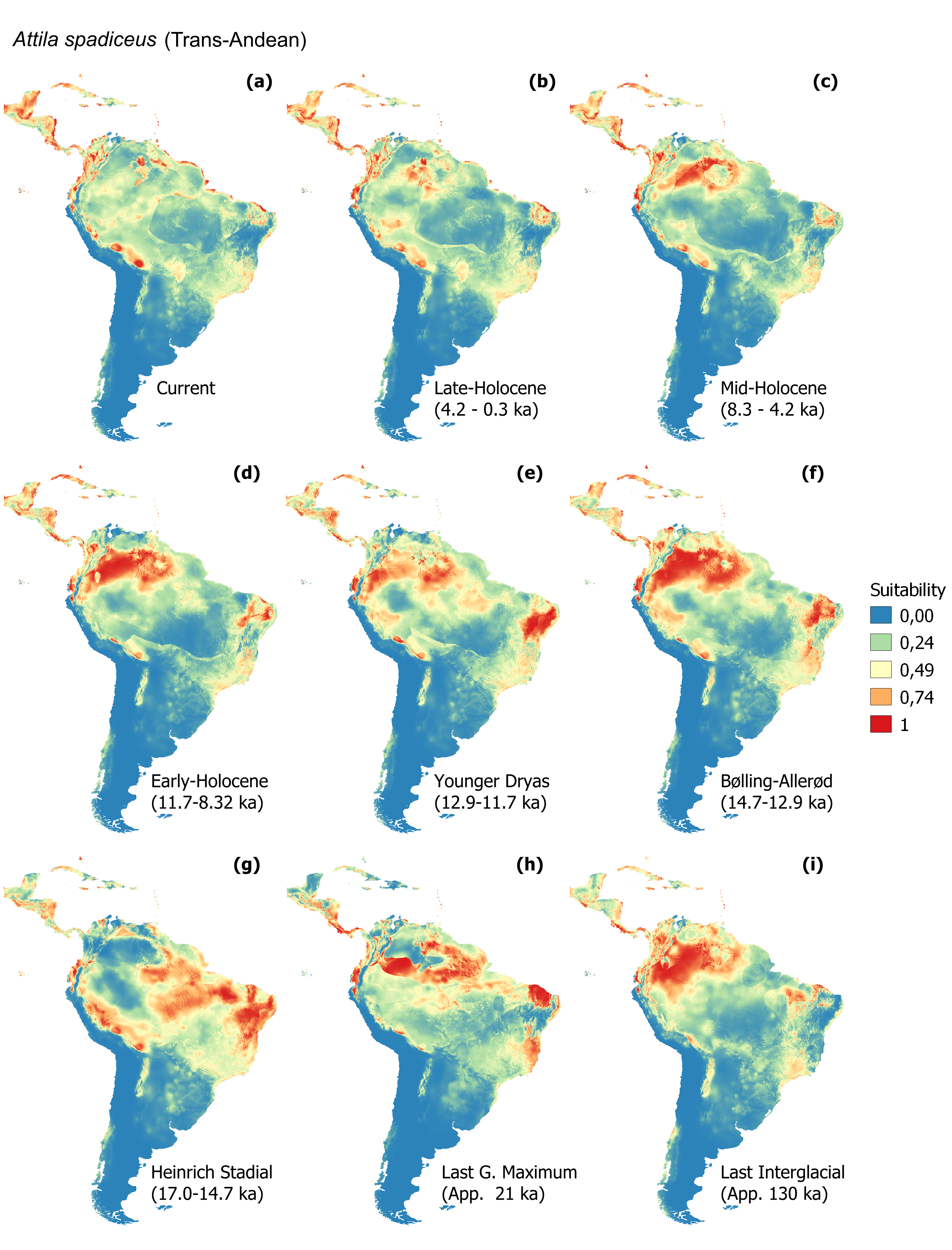


**APPENDIX 6. Figure S2.** (a) Niche overlap of the two lineages in the environmental space of the study area. The green shading represents the niche of the cis-Andean lineage, and the red shading represents the trans-Andean lineage. The blue shading corresponds to the overlap of the lineages. (b) Representation of the environmental variables (Table S4).


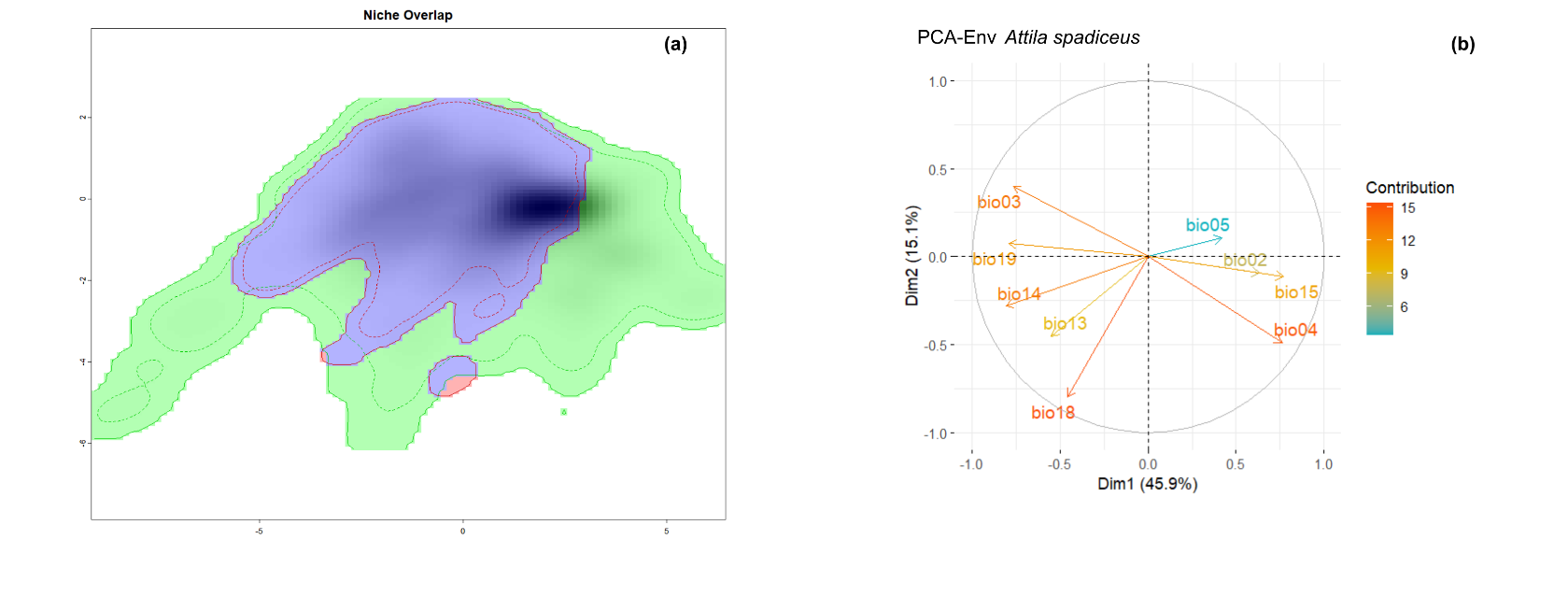


**APPENDIX 7. Figure S3.** Histogram of Schoener's D values for similarity and equivalence tests. (a) Similarity test comparing the trans- and cis-Andean lineages (D: 0.46; p: 0.0099). (b) Equivalence test comparing the trans- and cis-Andean lineages (D: 0.46; p: 0.0099).


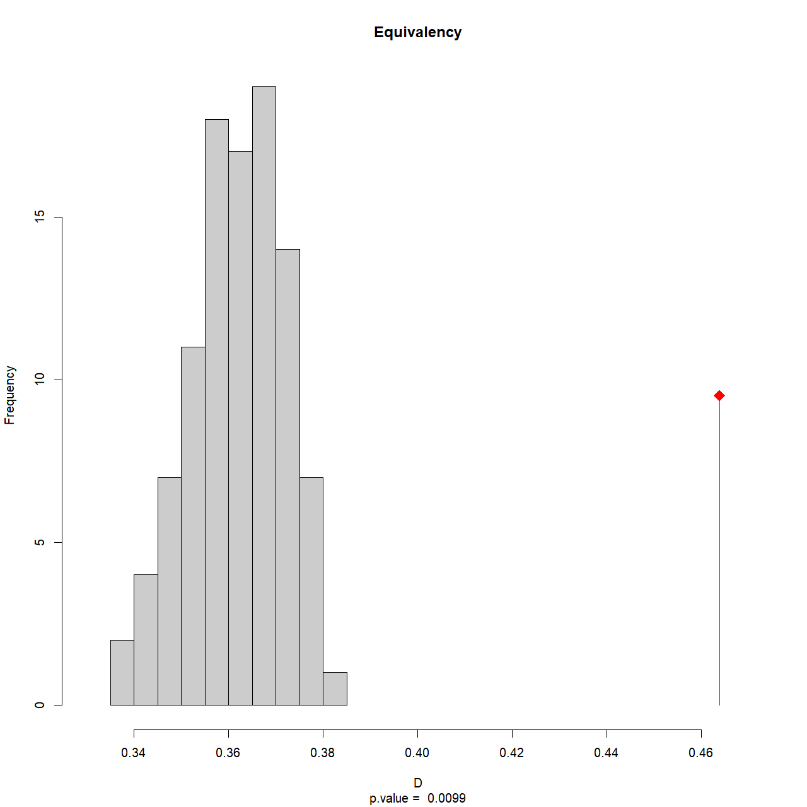

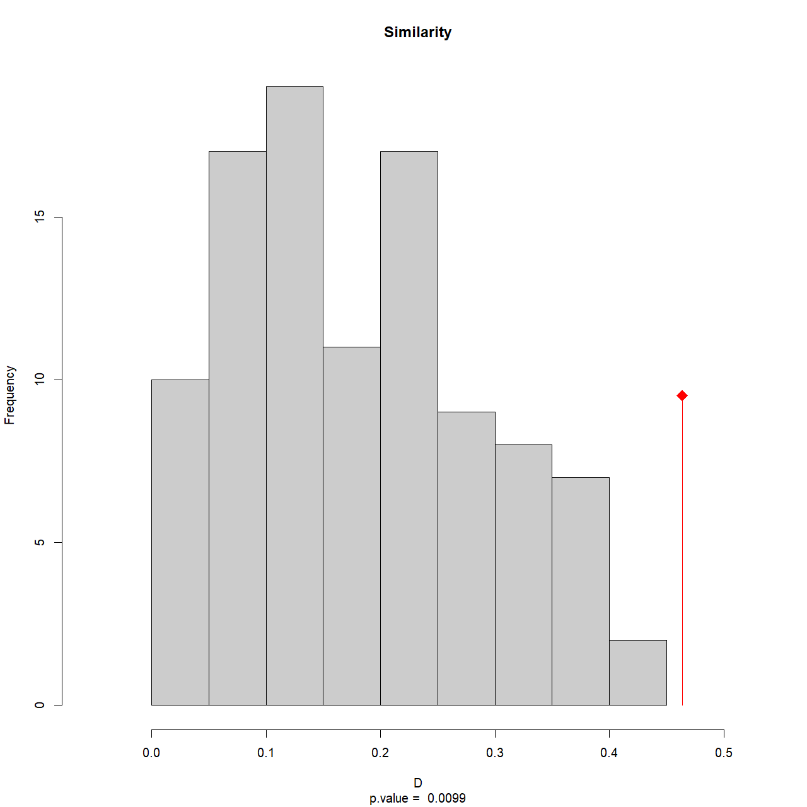

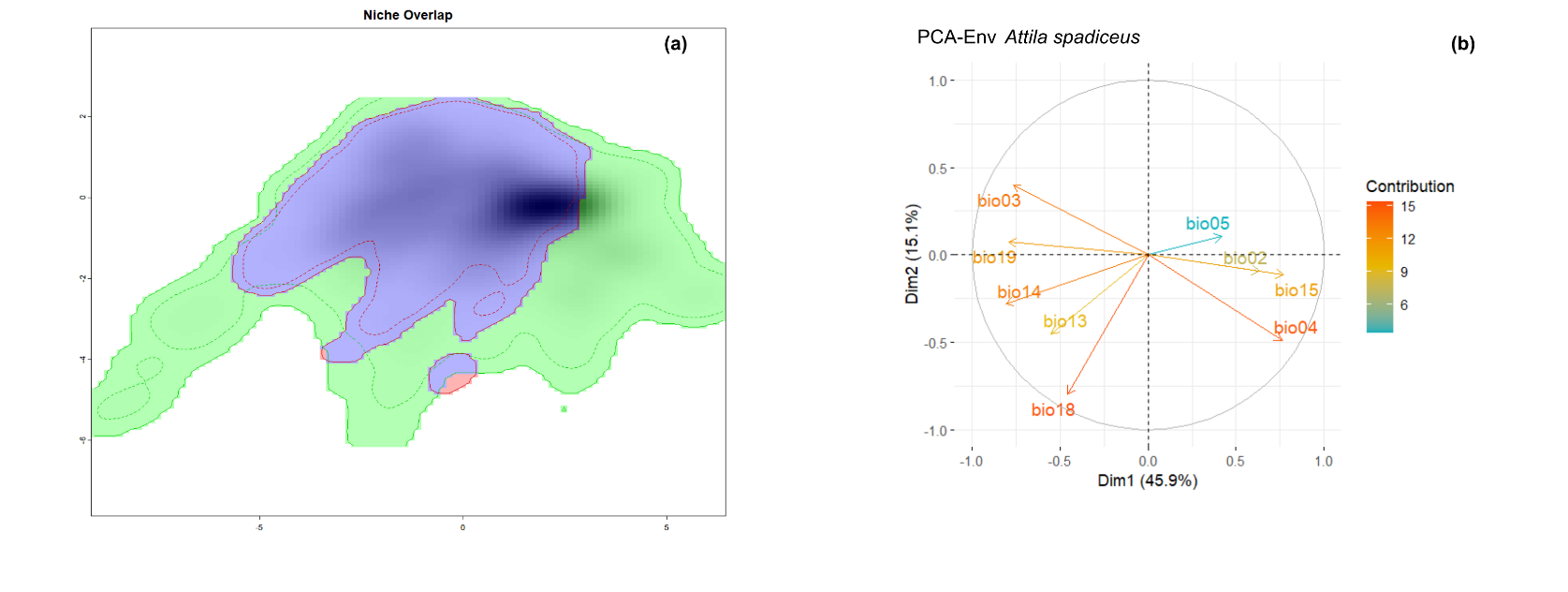

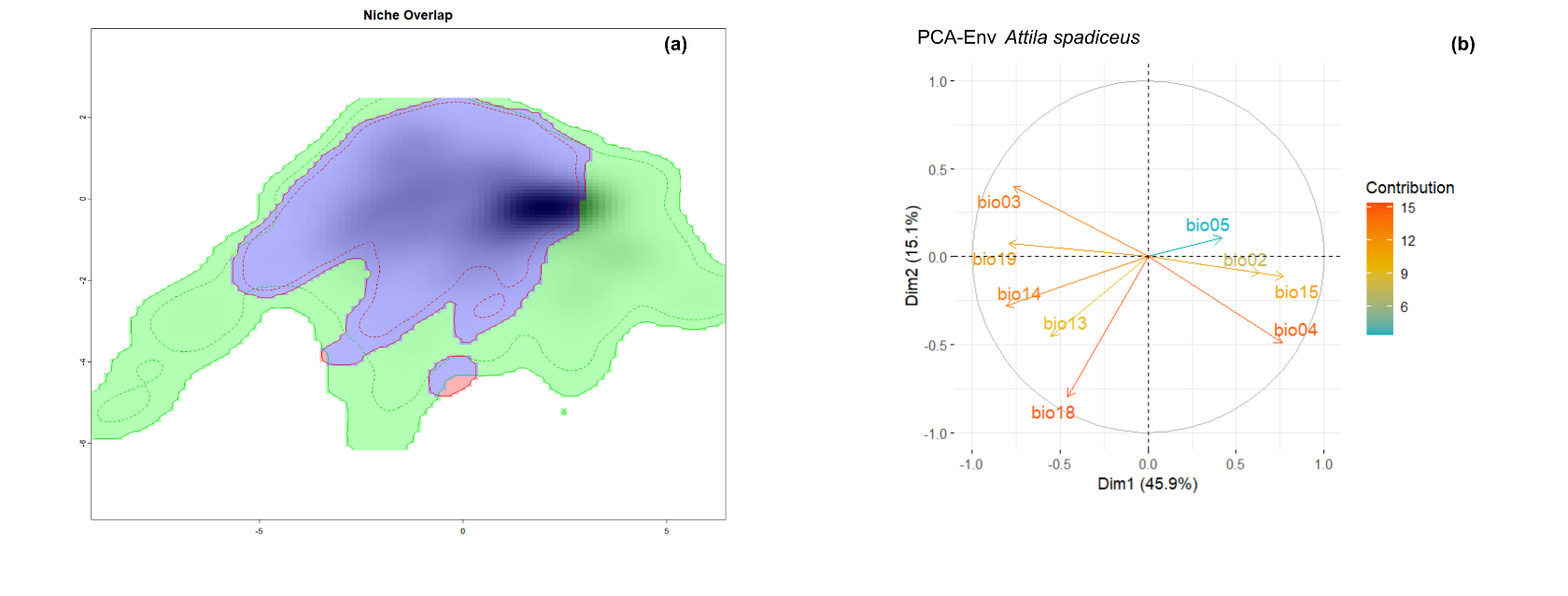

Supplement: Supplementary file 2 — Appendix S2. [file ECE3-15-e70331-s002.docx]
